# Synergizing Virtual Screening and Zebrafish Models to Identify Resveratrol-Derived Antiaging Polyphenols

**DOI:** 10.3390/ph18111630

**Published:** 2025-10-28

**Authors:** David Hernández-Silva, Cynthia Cabello, María Luisa Cayuela, Horacio Pérez-Sánchez, Francisca Alcaraz-Pérez

**Affiliations:** 1 Grupo de Telomerasa, Cáncer y Envejecimiento, Hospital Clínico Universitario Virgen de la Arrixaca, 30120 Murcia, Spain; 2 Instituto Murciano de Investigación Biosanitaria-Pascual Parrilla (IMIB-Pascual Parrilla), 30120 Murcia, Spain; 3 Structural Bioinformatics and High-Performance Computing Research Group (BIO-HPC), Computer Engineering Department, Universidad Catolica de Murcia (UCAM), 30107 Murcia, Spain; 4 Centro de Investigación Biomédica en Red de Enfermedades Raras (CIBERER), ISCIII, 28029 Madrid, Spain

**Keywords:** aging, short telomeres, chronic inflammation, virtual screening, zebrafish, resveratrol, polyphenols

## Abstract

**Background:** Telomere shortening and chronic inflammation are well-established hallmarks of aging and age-related diseases, often resulting in impaired cellular function. Identifying compounds with anti-aging potential is therefore crucial to promote healthy aging and extend lifespan. Virtual screening has emerged as a rapid and cost-effective strategy to assess the biological activity of large compound libraries. In parallel, the zebrafish (*Danio rerio*) model offers unique advantages for in vivo aging research and phenotypic screening. The integration of in silico and in vivo approaches has proven to enhance the efficiency and precision of therapeutic discovery. **Methods:** In this study, we combined ligand- and structure-based virtual screening to identify resveratrol-like polyphenols from the DrugBank database and evaluated their anti-aging effects in zebrafish models. **Results:** Among the top eight candidates, resveratrol and sakuranetin significantly improved telomerase-related parameters, while apigenin, genistein, and hesperetin exhibited notable anti-inflammatory activity. **Conclusions:** These findings underscore the value of combining computational and experimental models to accelerate the discovery of therapeutic agents targeting aging-related processes. The dual computational approach (pharmacophore similarity plus consensus docking) provided a robust prioritization pipeline directly validated in zebrafish assays.

## 1. Introduction

Aging is a complex biological process characterized by the progressive decline of physiological functions due to the accumulation of molecular, cellular, and tissues damage over time. This process increases susceptibility to a wide range of diseases, including diabetes, cardiovascular disorders, neurodegenerative conditions, and cancer [1]. Among the universally conserved hallmarks of aging are telomere shortening, the gradual loss of repetitive sequences at the ends of chromosomes, leading to chromosomal instability, replicative senescence and apoptosis [2,3]. Within the adaptive immune system, age-related telomere shortening contributes to immunosenescence, a state marked by cellular senescence and reduced replicative capacity [4]. This phenomenon is associated with lymphopenia, the progressive depletion of naïve T cells, and diminished T cell proliferation [5,6]. Despite the occurrence of immunosenescence, aged organisms often exhibit a chronic, low-grade inflammatory state known as inflammaging, characterized by elevated pro-inflammatory markers and persistent activation of the innate immune system, even in the absence of overt diseases [7].

Understanding the mechanisms underlying aging can inform the development of targeted therapies aimed at slowing or reversing age-related decline, ultimately promoting healthspan and longevity. Virtual screening (VS) has emerged as a powerful computational approach for identifying bioactive molecules [8]. Compared to traditional experimental methods, computational techniques for drug discovery, enhancement, and repositioning are faster more cost-effective and highly scalable, especially when consensus docking and pharmacophore modeling are combined to reduce false positives [9]. Once candidate molecules are identified in silico, preclinical studies are essential to evaluate their pharmacokinetic and therapeutic potential in animal models. Zebrafish larvae provide a particularly suitable high-throughput in vivo model due to the conservation of biological pathways and chemical responses between humans and zebrafish [10,11]. Integrating VS with zebrafish screening enables a streamlined drug discovery process, reducing the number of candidates requiring testing in mice or early-phase clinical trials. This approach offers an efficient, cost-effective, and accurate strategy for identifying potential therapeutics, particularly when combined with drug repositioning, to address challenges in anti-aging research [12].

Zebrafish have also become a valuable model for studying aging, as they display numerous hallmarks of aging analogous to those observed in humans [13,14]. Recently, a premature aging model caused by telomere shortening during the larval stage, termed the short telomere 2nd generation (ST2) model, was described. ST2 larvae exhibit absent telomerase activity, telomere shortening, oxidative stress, DNA damage, apoptosis, senescence, and premature death [15]. Another model, the Spint1a-deficient zebrafish, has been developed to study inflammaging. These larvae present chronic skin inflammation, neutrophil infiltration, keratinocyte hyperproliferation with aggregate foci, and impaired epithelial integrity [16,17]. Both models are highly suitable for drug screening, as the relevant phenotype manifests at the larval stage.

Resveratrol, a polyphenol abundant in grape skin and seeds, has been shown to possess antioxidant, anti-inflammatory, and anti-cancer properties [18]. Resveratrol activates the sirtuin family, the Nrf2 pathway and telomerase [19,20,21,22]. Furthermore, resveratrol also modulates transcriptional regulators to alleviate various age-related diseases, including inflammatory, cardiovascular and neurodegenerative disorders [23,24,25]. However, the poor bioavailability of resveratrol in humans has limited the translation of these findings into clinical benefits [26].

Considering its diverse biological activities, resveratrol was used as a reference molecule in this study for VS. Structurally related polyphenols were evaluated in vivo using zebrafish models of premature aging (ST2) and inflammaging (Spint1a-deficient). The goal was to identify polyphenols with improved bioavailability and enhanced efficacy. By combining a dual VS strategy (ligand-based pharmacophore modeling and consensus docking) with zebrafish assays, we aimed to systematically prioritize and experimentally validate novel compounds capable of modulating telomere function and inflammation in vivo, ultimately advancing the development of effective anti-aging interventions.

## 2. Results

### 2.1. In Silico Analysis for the Selection of the Top Ten Resveratrol-Derived Molecules

Given that resveratrol has been reported to activate SIRTUIN-5 [27], a mitochondrial isoform with a well-characterized structure, ligand-binding profile, and substrate specificity [28], several structure-based virtual screening (SBVS) calculations targeting this enzyme were performed. Initially, the interaction sites between the ligand and its receptor by using the crystal structure obtained from the Protein Data Bank (PDB ID: 4HDA) were analyzed. Next, multiple docking engines including LeadFinder (LF), AutoDock 4 (AD) and AutoDock Vina (AK) were employed to determine which tool most accurately reproduced the known interactions between resveratrol and SIRTUIN-5. Docking calculations were then performed against the DrugBank database (>10,000 compound) generating ranked lists of potential ligands for each doking engine. These results were integrated to produce a consensus set of 550 top-ranked compounds, ensuring a robust selection through cross-validation across the three independent docking platforms.

**Figure 1:**
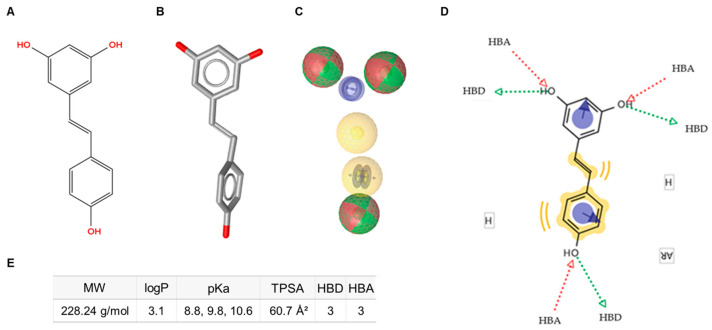
Schematic representation of the pharmacophore characteristics of trans-resveratrol. (**A**) Two-dimensional (2D) molecular structure. (**B**) Three-dimensional (3D) molecular structure. (**C**) Pharmacophore features: hydrogen-bond donors (HBD, green spheres), hydrogen-bond acceptors (HBA, red spheres), hydrophobic features (H, yellow spheres), and aromatic rings (AR, blue circles). (**D**) Diagram illustrating the interactive sites. (**E**) Annotation of key physicochemical properties of trans-resveratrol. MW: molecular weight; TPSA: topological polar surface area.

In parallel, a ligand-based virtual screening (LBVS) approach using LigandScout 4.4 was performed to generate a pharmacophore model of resveratrol (Figure 1A,B). The resulting pharmacophoric features included hydrogen-bond donors, hydrogen-bond acceptors, hydrophobic moieties, and aromatic rings (Figure 1C). This model provided a comprehensive template for subsequent screening steps by defining the key molecular interaction features for database comparison (Figure 1D).

**Figure 2:**
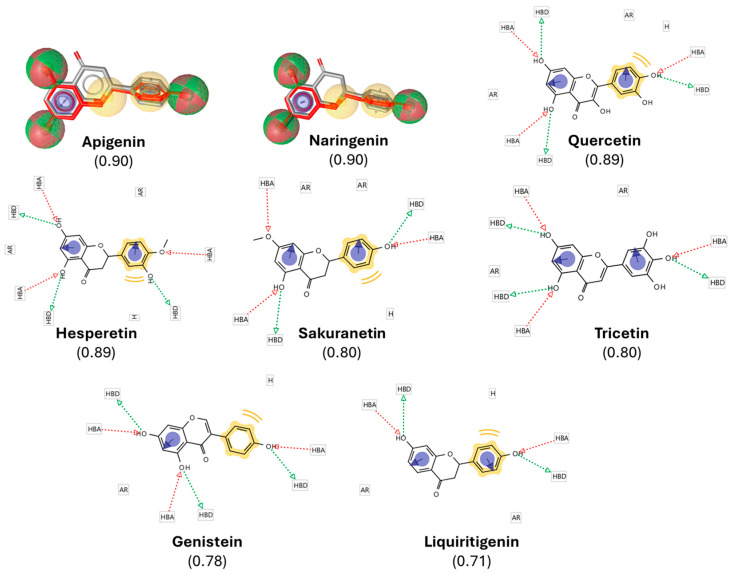
Pharmacophore-based selection of resveratrol-derived polyphenols. A pharmacophore model of resveratrol was generated in LigandScout 4.4, highlighting hydrogen-bond donors (HBDs, green spheres), hydrogen-bond acceptors (HBAs, red spheres), hydrophobic moieties (H, yellow spheres), and aromatic rings (ARs, blue circles). The model was used to screen the DrugBank database, yielding polyphenols with high structural and pharmacophoric similarity scores (0.71–0.90, indicated in brackets). The figure shows the overlay of resveratrol (gray/red) with representative top candidates, illustrating conservation of key pharmacophoric features. These similarities provided the rationale for selecting the compounds for subsequent docking and in vivo validation.

Following the LBVS, we obtained a comprehensive list of over 5000 compounds exhibiting both structural and pharmacophoric similarities to resveratrol. This dataset was ranked according to a similarity score, which quantitatively reflects the degree of correspondence between each compound and the target molecule in terms of structural alignment and pharmacophoric features. The similarity score ranged from 0 to 1, with 1 representing a perfect match and 0 indicating no similarity, as benchmarked against the DrugBank database. To ensure a stringent yet inclusive selection, a threshold of 0.7 was applied, thereby rotating compounds with a high degree of similarity to resveratrol while preserving structural diversity, a factor potentially critical for the discovery of novel bioactive molecules. Based on this criterion, 8 polyphenols with similarity scores ranging from 0.7 to 1 were identified (Figure 2). Cross-validation against the final protein-ligand docking results further confirmed that these 8 candidates were present among the top-ranked docking outputs.

### 2.2. In Silico Screening Predicts the Interaction Between Selected Resveratrol Derivatives and the Catalytic Subunit of Telomerase, TERT

**Figure 3:**
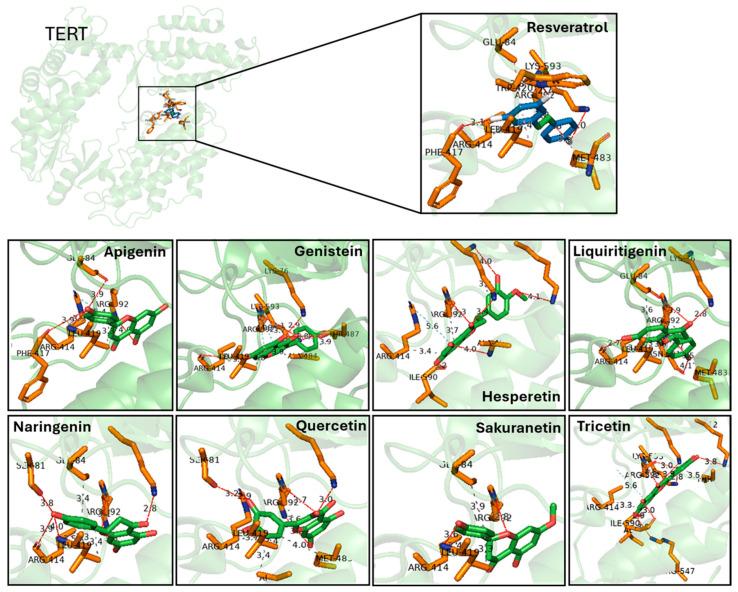
Predicted binding interactions between resveratrol-derived polyphenols and the catalytic subunit of human telomerase, TERT (PDB ID: 3DU6). TERT is shown in green cartoon representation, with interacting amino acid side chains in orange sticks. Each ligand is displayed in green sticks, while resveratrol is highlighted in the inset for comparison. Hydrogen bonds are indicated as dashed lines with distances in Å. Key residues involved in hydrogen bonding include Glu484, Lys592, Arg631, and Phe114, which are recurrently contacted across several ligands. Docking poses illustrate how different polyphenols occupy the catalytic pocket with overlapping orientations to resveratrol, supporting their predicted affinity. Distances represent top-scoring docking solutions obtained with consensus docking (AutoDock4, AutoDock Vina and LeadFinder).

Prior to evaluating the effect of the selected molecules in zebrafish in vivo models of aging, we performed a novel protein-ligand docking analysis to predict the interaction of these polyphenols with the catalytic subunit of telomerase, TERT. The crystal structure of TERT (PDB ID: 3DU6) was used as a reference, and the three docking engines—AD, LF and AK—were employed to analyze the binding of each polyphenol, including resveratrol. The AD docking calculations revealed that all predicted protein-ligand interactions occurred in clusters 1, 2 and 3, which represent binding modes considered highly likely to occur in vivo (Appendix A Table A1). Based on these docking calculations, Figure 3 illustrates the predicted interaction region of TERT with the different polyphenols.

In summary, by applying a VS strategy based on both structural and pharmacophoric similarity to resveratrol, we identified 8 natural polyphenolic compounds with predicted binding affinity to TERT and potential anti-aging activity from an initial library of more than 11,000 candidates. These findings highlight the selected compounds as promising candidates for subsequent in vivo evaluation in zebrafish models of premature aging and inflammaging.

### 2.3. Resveratrol and Sakuranetin Improve Telomeric Health, with Resveratrol Further Enhancing ST2 Larval Longevity

**Figure 4:**
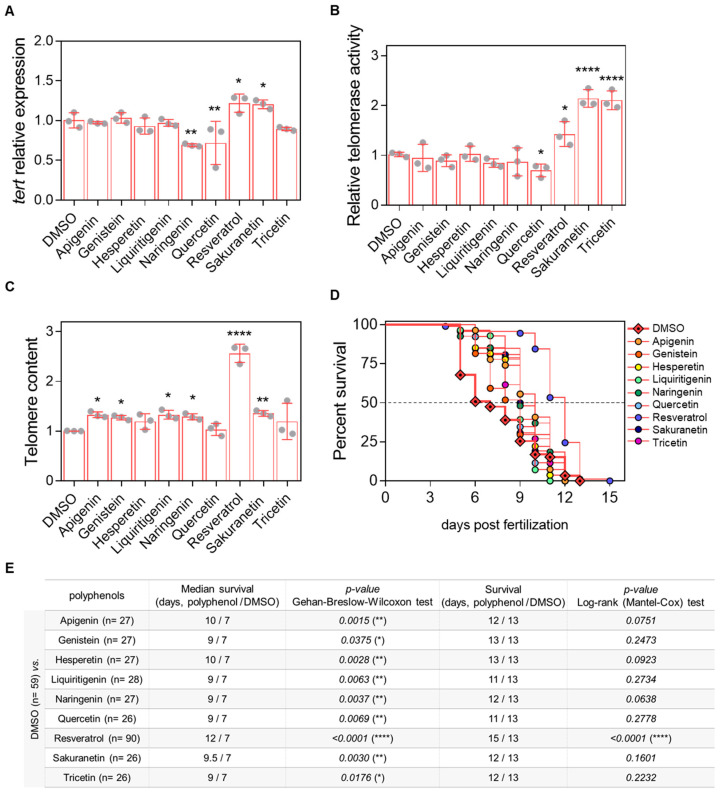
In vivo evaluation of the telomeric effect of resveratrol-derived polyphenols in the prematurely aged ST2 model. (**A**) *tert* mRNA levels were quantified in 3-dpf zebrafish larvae by real-time RT-qPCR and normalized to *rps11*. (**B**) Telomerase activity was measured in 3-dpf zebrafish larvae using 0.5 µg of protein extract. (**C**) Telomere length was assessed in 3-dpf zebrafish larvae by qPCR using 60 ng of gDNA and expressed as telomere content relative to the single-copy gene *rps11*. Bars represent the mean ± SD of 3 independent experiments (N = 3) with 20–25 pooled larvae each (n = 20–25), relative to DMSO control. * *p* < 0.05, ** *p* < 0.01, **** *p* < 0.0001, determined by one-way ANOVA followed by uncorrected Fisher’s LSD test. (**D**) Kaplan–Meier survival curves of ST2 larvae under different treatments. (**E**) Summary of survival metrics. The table indicates sample size (in brackets), survival duration (days), and associated *p*-values for each treatment condition.

**Figure A1:**
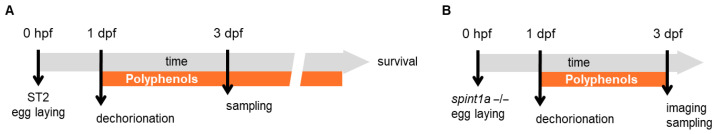
General workflow for polyphenol treatment of the ST2 larvae (**A**) and the *spint1a−/−* larvae (**B**). At 1 dpf, zebrafish embryos were manually dechorionated and treated with polyphenols or vehicle alone (DMSO 0.1%) for a period of 2 days. For survival studies, the treatment was renewed every 48 h.

**Figure A2:**
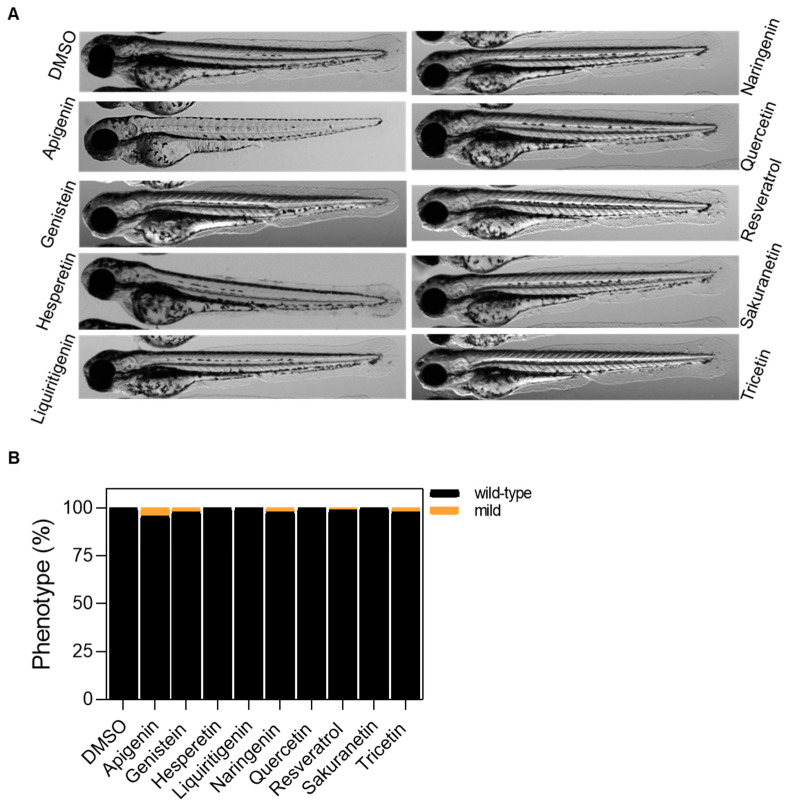
Tolerability evaluation of polyphenols in wild-type zebrafish larvae after 48 h of treatment. (**A**) Representative images of 72 hpf larvae after 48 h of treatment with polyphenols. (**B**) Classification of 72 hpf larvae by phenotype. Differences are not statistically significant according to Two-way ANOVA followed by Sidak’s multiple comparison test. In all cases, the working dose is 10 μM, except for genistein, which is 5 μM. Scale bar: 500 μm.

After establishing the working dose and treatment conditions (Appendix A Figure A1 and Figure A2), prematurely aged ST2 larvae were used as an in vivo model to evaluate the effects of resveratrol-derived polyphenols on telomere-related parameters, including *tert* mRNA levels, telomerase activity, and telomere length at 3 days post-fertilization (dpf). Among the compounds tested, only resveratrol and sakuranetin increased *tert* mRNA levels, whereas naringenin and quercetin had a negative effect (Figure 4A). Regarding telomerase activity, resveratrol, sakuranetin and tricetin significantly enhanced enzymatic activity, while quercetin treatment resulted in a decrease (Figure 4B). Assessment of telomere length revealed a modest positive effect of apigenin, genistein, liquiritigenin, naringenin and sakuranetin with resveratrol showing the most pronounced increase (Figure 4C). Finally, survival analysis of ST2 larvae demonstrated that resveratrol was the only polyphenol capable of slightly extending survival by 15.4%, while the other compounds produced only moderate increases in mean survival (Figure 4D,E).

### 2.4. Apigenin, Genistein and Hesperetin Show Both Anti-Inflammatory and Antioxidant Properties

**Figure 5:**
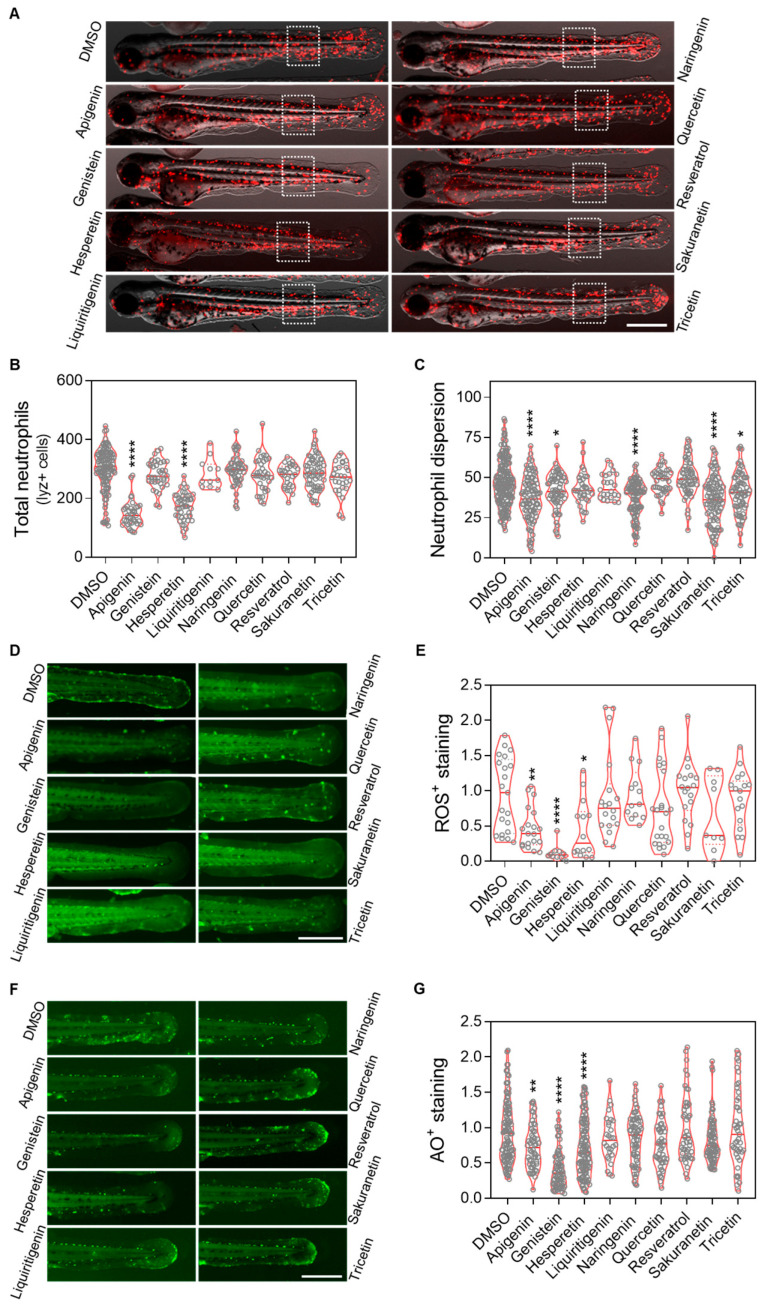
In vivo evaluation of the anti-inflammation and antioxidant effect of resveratrol-derived polyphenols in the *spint1a*−/− inflammaging model. (**A**) Representative images of *lyz:dsRed2* in *spint1a* knockout zebrafish larvae from each treatment group. (**B**) Quantification of total neutrophils counts. (**C**) Quantification of neutrophils located outside the caudal hematopoietic tissue (CHT) within the indicated region of interest (ROI, discontinuous white squares) after 2 days of treatment. Neutrophils are labeled in red. (**D**) Representative images of ROS staining in *spint1a−/−* larvae from each treatment group. (**E**) Quantification of the cellular oxidative stress levels in the tail after 2 days of treatment. (**F**) Representative images of acridine orange (AO) staining in *spint1a−/−* larvae from each treatment group. (**G**) Quantification of cell death levels in the tail after 2 days of treatment. Violin plots display the distribution with the media indicated by a red horizontal line, overlaid with raw data points representing individual larvae from two to four independent experiments (N = 2–4). Statistical significance: * *p* < 0.05; ** *p* < 0.01; **** *p* < 0.0001, determined by one-way ANOVA followed by Dunnett’s multiple comparison test. Scale bar: 500 μm.

To evaluate the anti-inflammatory and antioxidant effects of resveratrol-derived polyphenols in vivo, the Spint1a-deficient zebrafish inflammaging model was used. 1-dpf larvae were treated with the candidate compounds for 48 h (Appendix A Figure A1B). We observed that apigenin, genistein, naringenin, sakuranetin and tricetin mitigated the neutrophil dispersion phenotype in a transgenic line with labeled neutrophils (*lyz:DsRED2*) (Figure 5C), although only apigenin and hesperetin significantly reduced the total number of neutrophils (Figure 5D). Regarding oxidative stress, apigenin, genistein and hesperetin were able to decrease reactive oxygen species (ROS) levels; however, contrary to expectations, resveratrol did not show any antioxidant effect (Figure 5F). Finally, analysis of cell death rates confirmed the protective effects of apigenin, genistein and hesperetin, consistent with the previous observations (Figure 5G).

## 3. Discussion

Understanding the fundamental mechanisms of aging and their connection to chronic age-related diseases is a major focus in biomedical research, with the ultimate goal of delaying their onset and extending healthy lifespan [29]. In this context, the search for new therapeutic agents to counteract aging and improve overall health is especially relevant. However, the path from discovery to effective treatment remains challenging. During clinical development, many compounds are discontinued, with attrition rates reaching up to 96%. This high failure rate is often due to factors such as poor ADMET (Absorption, Distribution, Metabolism, and Excretion) profiles and the use of poorly characterized research models. These limitations not only delay the development of new therapies but also consume significant financial resources and human effort, ultimately slowing progress in improving patient outcomes and highlighting the need for robust, well-characterized models in aging research [30].

VS has become a key in silico strategy for identifying new therapeutic compounds, offering speed and cost-effectiveness, thanks to the availability of free academic tools and docking programs [8]. Its utility lies in its ability to drastically reduce the number of molecules requiring experimental testing from millions to only tens. In our study, we applied a dual approach combining consensus docking across three engines (AutoDock4, AutoDock Vina, LeadFinder) with pharmacophore-based screening using LigandScout, which increases robustness compared to single-method VS.

Resveratrol, a natural antioxidant approved by the FDA for reducing cellular damage and promoting healthspan, has shown beneficial effects in the ST2 premature aging model [15], making it a suitable reference scaffold for identifying similar compounds through VS. Beyond its direct impact on telomerase-related parameters, resveratrol interacts with several molecular targets that may contribute to its anti-aging effects. Among the most studied is sirtuin 1 (SIRT1), an NAD^+^-dependent deacetylase involved in cellular stress responses, DNA repair, and metabolic regulation—processes closely linked to aging [31,32]. Resveratrol also modulates estrogen receptors, AMP-activated protein kinase (AMPK), and key signaling pathways associated with oxidative stress and inflammation, such as MAPKs and PI3K/Akt [33]. These off-target interactions may help explain its pleiotropic effects and broaden its therapeutic potential beyond telomerase modulation. Altogether, these mechanisms support its inclusion in our screening pipeline and reinforce the value of multi-target approaches when evaluating anti-aging compounds.

Using the structural and pharmacophoric features of resveratrol as a template, we identified eight natural antioxidant compounds with potential anti-aging properties from a library of more than 11,000 candidates (Figure 2). Compounds were prioritized using pharmacophore similarity (≥0.7) and top consensus docking ranks across the three engines. Notably, all eight compounds were predicted to interact with TERT (Figure 3), potentially enhancing its biological activity. Binding energies ranged from −7.2 to −9.1 kcal/mol, with recurrent hydrogen bonding to Lys592 and Arg631, supporting the plausibility of these interactions. These candidates were subsequently selected for in vivo evaluation in zebrafish models of aging and inflammaging. Importantly, all of them have been previously described as having antioxidant and anti-inflammatory properties, and their administration has been shown to mitigate several age-related phenotypes, including oxidative stress, chronic inflammation, impaired proteostasis, and cellular senescence—features closely associated with increased risk of age-related diseases [34].

**Figure 6:**
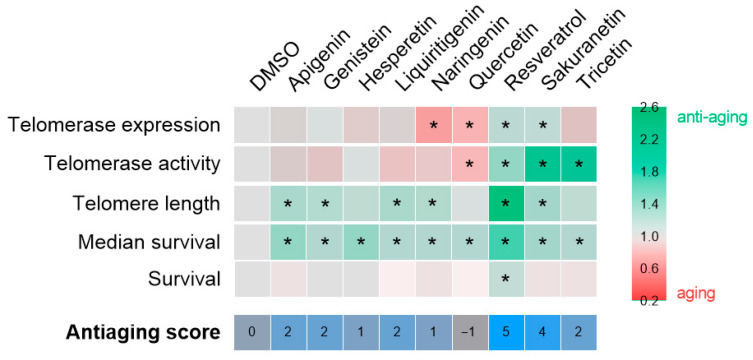
Compilation anti-aging potential of polyphenolic compounds in the context of telomere shortening. The heat map illustrates the fold change of each compound relative to the vehicle, DMSO, for each of the parameters evaluated. The asterisk denotes statistical significance. The anti-aging score, which is determined by the number of hits achieved, is displayed at the bottom of the figure.

After confirming their safety, the anti-aging potential of the selected compounds was tested in the ST2 premature aging model, characterized by telomere shortening (Figure 4). The results are summarized in Figure 6. Contrary to the initial hypothesis, sakuranetin was the only resveratrol-derived polyphenol to exhibit a comparable effect to resveratrol. However, it did not extend the survival of ST2 larvae, likely due to suboptimal dosing. Treatment with the remaining candidates generally improved the average survival, although the underlying mechanisms remain unclear. A notable advantage of the ST2 model, which features genotype heterogeneity, is its ability to reveal both Tert-dependent and Tert-independent effects. In this context, the observed benefits of apigenin, genistein, liquiritigenin, and naringenin may be linked to alternative telomere lengthening mechanisms. Conversely, the effects of hesperetin, quercetin, and tricetin appear to be independent of telomere length, underscoring the need to investigate additional aging-related biomarkers. Overall, sakuranetin (antiaging score = 4) stands out among the candidates and represents the most promising polyphenol for validation in a single-genotype model, enabling a more detailed examination of its mechanism of action.

A persistent challenge in drug discovery is the limited correlation between in silico predictions and in vivo outcomes. In theory, this gap could be narrowed by incorporating additional computational analyses—such as molecular dynamics simulations, detailed ADMET profiling, or machine learning–based scoring functions. However, these approaches are time-consuming and resource-intensive, and still fall short of capturing the biological complexity of living systems. For this reason, we believe that the hybrid strategy proposed here—initial in silico selection followed by rapid in vivo validation—not only enables faster prioritization of promising candidates, but also facilitates the identification of active compounds within a biologically relevant timeframe [12].

**Figure 7:**
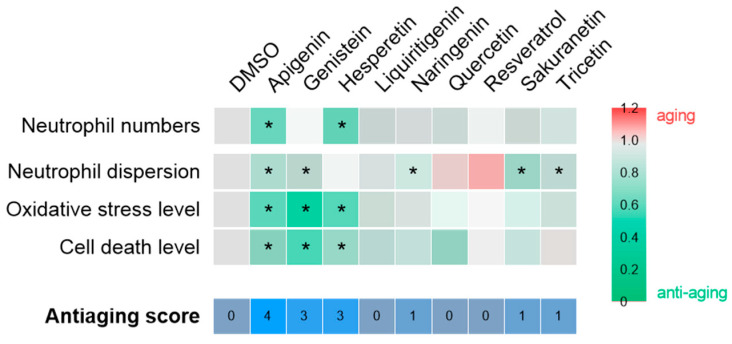
Compilation of anti-aging potential of polyphenolic compounds in the context of chronic inflammation. The heat map illustrates the fold change of each compound relative to the vehicle, DMSO, for each of the parameters evaluated. The asterisk indicates statistical significance. The anti-aging score, which is determined by the number of successful hits, is displayed at the bottom of the figure.

In the context of chronic inflammation, the effects of polyphenols on the inflammaging phenotype of Spint1a-deficient larvae were assessed (Figure 5). As shown in Figure 7, apigenin (anti-aging score = 4), followed by genistein and hesperetin (anti-aging score = 3), showed the greatest anti-aging potential under inflammatory conditions. These compounds effectively reduced neutrophil number and/or dispersion, as well as oxidative stress and cell death. Consequently, apigenin, genistein, and hesperetin emerge as promising candidates for further investigation as potential interventions to prevent aging associated with chronic inflammation. In contrast, the remaining compounds—including resveratrol—showed no significant efficacy in ameliorating this phenotype. The lack of effect of resveratrol is particularly noteworthy, given its established role as a reference compound with recognized antioxidant properties. Based on the data obtained in this and previous studies, the antioxidant capacity of resveratrol appears to be limited to contexts where oxidative stress is a consequence of telomere shortening, as observed in the ST2 model [15], but not in the *spint1a*−/− inflammaging model [35].

In summary, our findings highlight the potential of several polyphenols as anti-aging agents. Resveratrol and sakuranetin emerged as the most promising candidates for mitigating telomere shortening, while apigenin, genistein, and hesperetin showed the greatest efficacy in reducing chronic inflammation. Further studies in adult aging models are needed to clarify their mechanisms of action and assess their long-term therapeutic potential. Expanding these investigations could open new therapeutic avenues for improving quality of life in aging populations and for the treatment of chronic age-related diseases such as arthritis, cardiovascular conditions, and neurodegenerative disorders.

## 4. Materials and Methods

### 4.1. Molecule File Preparation

For protein preparation, sirtuins complexed with ligands obtained from the RCSB Protein Data Bank (PDB) (PDB ID: 4HDA). Utilizing PyMOL version 2.0, the ligand was extracted, and the molecules were subsequently converted to .mol2 format in MOE version 1.3 to ensure compatibility with downstream docking tools. The sirtuin structures were prepared by removing crystallographic water molecules and adding polar hydrogens, and then converted to .pdbqt format using AutoDock Tools version 4.2, enabling their use in molecular docking workflows.

### 4.2. Ligand Database

DrugBank version 5.1.1 (July 2018 release) was used as the source for compound data, provided in both .mol2 and .pdbqt formats [36]. The dataset included 11,538 entries, comprising approximately 2700 approved small-molecule drugs, 1500 biologics (including proteins, peptides, vaccines, and allergenic extracts), 132 nutraceuticals, and over 6700 experimental compounds. This chemically diverse library was selected to enable a broad and representative screening of bioactive molecules with potential anti-aging effects. To ensure structural integrity and avoid redundancy, around 5–10% of the entries (≈550–1100 compounds) were excluded due to incomplete molecular data, salt forms, or duplication.

### 4.3. Ligand-Based Virtual Screening (LBVS)

The pharmacophore model for the target molecule was generated using LigandScout 4.4, allowing the selection of compounds based on scoring and feature similarity with reference molecules. Key features included pharmacophoric elements critical to bioactivity prediction. The performance of LigandScout 4.4 facilitated the efficient identification of candidate compounds, ranked by pharmacophore fit score (0–1 scale), which were subsequently shortlisted for further evaluation. A similarity threshold of ≥0.7 (based on the Tanimoto coefficient) was selected in line with previous studies showing that compounds with similarity values above this level often share common chemical scaffolds or exhibit related biological activities [37,38,39].

### 4.4. Animals

Wild-type AB zebrafish (*Danio rerio*) were obtained from the Zebrafish International Resource Centre (ZIRC) and mated, staged, raised and processed using standard procedures. Comprehensive husbandry and environmental protocols are available on protocols.io (https://dx.doi.org/10.17504/protocols.io.mrjc54n, accessed on 1 September 2025) [40]. The ST2 zebrafish line was maintained in our laboratory and has been described previously [15]. The mutant zebrafish line *spint1ahi2217Tg*/*hi2217Tg* (*spint1a*−/−) was isolated from an insertional mutagenesis screen [16], and the transgenic line *Tg(lyz:dsRED2)^nz50^* (*lyz:dsRED2*, for simplicity) has been previously characterized [41].

Sample size decisions were based on prior zebrafish screening studies and the effect sizes typically detectable under standard conditions, in accordance with the Refinement principle. To mitigate the potential for selection bias, the larvae were randomly assigned to the experimental groups. Molecular assays were conducted using pools of 20–25 larvae per group. Survival and imaging assays were performed on individual larvae. The precise sample counts for each condition can be found in the Supplementary Primary Data Excel file.

### 4.5. Drug Treatment

Polyphenols were resuspended in DMSO, and an initial dose of 10 µM was tested in wild-type larvae, consistent with standard high-throughput screening concentrations. At 24 h post-fertilization (hpf), zebrafish embryos were manually dechorionated and treated with polyphenols or vehicle (0.1% DMSO) for a period of 48 h (Appendix A Figure A1). Any injured or non-viable individuals were excluded from analysis. Larval morphology and physiology were monitored to assess compound tolerability (Appendix A Figure A2). Following this evaluation, a working dose of 10 µM was applied to all polyphenols, except for genistein, which was tested at 5 µM due to toxicity observed at 10 µM.

The treatments were coded by one of the authors and administered in a fixed, pre-defined order. Outcome assessment was conducted blindly to reduce bias.

### 4.6. Gene Expression Analysis

Total RNA was extracted after mechanical homogenization using TRIzol reagent (Thermo Fisher Scientific, Waltham, MA, USA) and the Direct-zol RNA Miniprep Kit (Zymo Research, Irvine, CA, USA), following the manufacturer’s instructions. On-column DNase I treatment (RNase-free; Qiagen, Germantown, MD, USA) was performed to eliminate genomic DNA (gDNA) contamination. First-strand complementary DNA (cDNA) was synthesized using the SuperScript™ VILO™ cDNA Synthesis Kit (Invitrogen, Carlsbad, CA, USA), according to the manufacturer’s protocol. Quantitative PCR (qPCR) was carried out on a StepOnePlus instrument (Applied Biosystems, Foster City, CA, USA) using SYBR^®^ Premix Ex Taq™ (Perfect Real Time; Takara, Toshima-ku, Tokyo) and primers listed in Appendix A Table A2. Cycling conditions were 95 °C for 30 s, followed by 40 cycles of 95 °C for 5 s and 60 °C for 20 s, and a final melting curve step. Expression levels were normalized to the ribosomal protein S11 single-copy gene (*rps11*) and calculated using the comparative Ct method (2^−ΔΔCt^). All reactions were performed in triplicate.

### 4.7. Telomerase Activity Assay

After mechanical homogenization, proteins were extracted using ice-cold 3-[(3-cholamidopropyl)dimethylammonio]-1-propanesulfonate (CHAPS) lysis buffer (Millipore). Quantitative Telomere Repeat Amplification Protocol (qTRAP) was performed with 0.5 mg of protein extracts and primers listed in Appendix A Table A2, as described previously [42]. Negative control included 1 μg of RNase at 37 °C for 20 min to confirm assay specificity. A standard curve was constructed using a 1:10 dilution series of telomerase-positive HeLa cell extract, resulting in y = 23.802–3.2295x. Relative Telomerase Activity (RTA) was calculated as RTA = 10^(Ct sample-gint)/slope^.

### 4.8. Telomere Length Measurement

gDNA was extracted using the Wizard Genomic DNA Purification Kit (Promega, Madison, WI, USA). Telomere length was measured by quantitative PCR (qPCR) following established protocols [43], using primers listed in Appendix A Table A2 and 60 ng of gDNA per reaction. Reactions were performed on an ABI PRISM 7700 instrument (Applied Biosystems) with TB Green reagents (Applied Biosystems). Cycling conditions were 95 °C for 15 min, followed by 40 cycles of 95 °C for 15 s and 54 °C for 2 min, and a final step of 95 °C for 15 s, 60 °C for 1 min, and 95 °C for 15 s. Telomeric content relative to the single-copy *rps11* gene (T/S ratio) was calculated using the comparative Ct method (2^−ΔΔCt^). All reactions were performed in triplicate.

### 4.9. Survival Analysis

For survival analysis, 25–30 larvae per group were used and treatment was renewed every 48 h (Appendix A Figure A1A). Zebrafish larvae were monitored every 24 h for clinical signs of disease and mortality under a Leica M150C magnifying glass equipped with a digital camera (DMC 5400, Leica, Wetzlar, Germany).

### 4.10. Imaging of Zebrafish Larvae

At 3 days post-treatment (3 dpt), larvae were imaged using a Leica M205 FA fluorescence magnifying glass equipped with a DFC 365 FX digital camera, employing green and red fluorescent filters.

### 4.11. Neutrophil Counts

At 3 dpf, zebrafish larvae were anesthetized with 200 mg/mL buffered tricaine and imaged in vivo. Images were analyzed to quantify neutrophils number and distribution within a defined ROI.

### 4.12. Reactive Oxygen Species (ROS) Staining

Oxidative stress was assessed by measuring H_2_O_2_ release using the live-cell fluorogenic substrate, acetyl-pentafluorobenzene sulfonyl fluorescein (Cayman Chemical, Ann Arbor, MI, USA) [44,45]. Briefly, embryos at 72 hpf were incubated in 50 μM substrate in 1% DMSO for 1 h in a 96-well plate. Larvae were imaged as detailed above, and fluorescence intensity within a tail ROI was quantified using ImageJ version 1.52.

### 4.13. Acridine Orange (AO) Staining Assay

Cell death was evaluated using acridine orange (AO) staining according to the ZFIN ProtocolWiki (https://zfin.atlassian.net/wiki/spaces/prot/overview, accessed on 1 September 2025). Briefly, 3 dpf larvae were incubated in E3 medium containing 10 mg/mL AO (Sigma A6014, St. Louis, MO, USA) for 30 min at room temperature. Larvae were washed three times for 10 min each and imaged in tricaine-containing egg water (200 µg/mL). AO-positive cells were quantified from the images.

### 4.14. Statistical Analysis

Statistical analyses were conducted using GraphPad Prism version 8. Normality was assessed prior to hypothesis testing. Comparisons among more than two groups were analyzed by one-way ANOVA followed by either uncorrected Fisher’s LSD or Dunnett’s multiple comparison post hoc tests and two-way ANOVA followed by Sidak’s multiple comparison test. Survival curves were analyzed using both Gehan-Breslow-Wilcoxon and log-rank (Mantel–Cox) tests. A *p*-value < 0.05 was considered statistically significant. Detailed information is provided in the figure legends.

## 5. Conclusions

This study demonstrates the utility of integrating virtual screening with zebrafish in vivo models to efficiently identify natural compounds with anti-aging potential. Using resveratrol as a reference, we implemented a dual ligand- and structure-based virtual screening pipeline, combining pharmacophore modeling with consensus docking across three engines, to interrogate a large polyphenolic library. Eight structurally similar candidates were identified, exhibiting favorable docking interactions with the catalytic subunit of telomerase (TERT), with predicted binding free energies in the range of −7.2 to −9.1 kcal/mol and recurrent interactions with residues Lys592 and Arg631, suggesting potential roles in telomere maintenance. Functional validation in zebrafish models of premature aging (ST2) and chronic inflammation (*spint1a−/−*) revealed diverse, compound-specific biological effects. Resveratrol and sakuranetin were the most effective agents in restoring telomere integrity, while apigenin, genistein and hesperetin demonstrated strong antioxidant and anti-inflammatory activity, reducing neutrophil infiltration, oxidative stress, and cell death.

Overall, these findings validate the effectiveness of combining in silico screening with zebrafish-based functional assays for rapid and cost-effective discovery of anti-aging agents. The selected polyphenols represent promising candidates for mechanistic studies and preclinical development targeting aging and age-associated disorders.

## Data Availability

The raw data supporting the conclusions of this article are contained within the article or Supplementary Material.

## Supplementary Materials

The following supporting information can be downloaded at: https://www.mdpi.com/article/10.3390/ph18111630/s1.

## Abbreviations

The following abbreviations are used in this manuscript:

AD	AutoDock 4
AK	AutoDock Vina
AMPK	AMP-activated protein kinase
AO	Acridine Orange
AR	Aromatic Ring
CHAPS	3-[(3-Cholamidopropyl)dimethylammonio]-1-propanesulfonate
dpf	days post-fertilization
cDNA	complementary DNA
gDNA	genomic DNA
H	Hydrophobic moiety
HBA	Hydrogen Bond Acceptor
HBD	Hydrogen Bond Donor
hpf	hours post-fertilization
LBVS	Ligand-Based Virtual Screening
LF	LeadFinder
mRNA	messenger RNA
MW	Molecular Weight
PDB	Protein Data Bank
qPCR	quantitative Polymerase Chain Reaction
qTRAP	quantitative Telomerase Repeated Amplification Protocol
ROI	Region Of Interest
ROS	Reactive Oxygen Species
*rps11*	ribosomal protein S11 encoding zebrafish gene
RTA	Relative Telomerase Activity
SD	Standard Deviation
SIRT1	Sirtuin 1
ST2	Short Telomere 2nd round
*tert*	telomerase reverse transcriptase encoding zebrafish gene
Tert	Telomerase reverse transcriptase, zebrafish protein
TERT	TElomerase Reverse Transcriptase, human protein
TPSA	Topological Polar Surface Area
VS	Virtual Screening
ZFIN	ZebraFish Information Network
2D	Two-dimensional
3D	Three-dimensional

## Appendix A

**Table A1:** Prediction of the interaction between polyphenols and TERT based on AD docking. The conserved amino acids are shown on the left side of the table. X marks the amino acids where the protein-ligand interaction occurs. The interaction groups and energies are shown at the bottom.

Docking	AD	AD	AD	AD	AD	AD	AD	AD	AD
Candidates	Apigenin	Genistein	Hesperetin	Liquiritigenin	Naringenin	Quercetin	Resveratrol	Sakuranetin	Tricetin
414 Arg	X	X	X	X	X	X	X	X	X
419 Leu	X	X	X	X	X	X	X	X	
480 Ala						X			X
592 Arg	X	X	X	X	X	X		X	X
76 Lys		X	X	X	X	X			X
84 Glu	X			X	X		X	X	
483 Met				X			X		
547 Arg									X
593 Lys		X							X
Cluster	3	2	2	1	3	2	3	2	2
Energy	−7.76	−7.73	−8.92	−8.29	−7.69	−9.12	−6.81	−7.59	−7.85

**Table A2:** Primers used in this study.

Name	Sequence 5′→3′	Application
*tert*-Fw	CGGTATGACGGCCTATCACT	gene expression
*tert*-Rv	TAAACGGCCTCCACAGAGTT	gene expression
ACX	GCGCGGCTTACCCTTACCCTTACCCTAACC	telomerase activity
TS	AATCCGTCGAGCAGAGTT	telomerase activity
*telom*-Fw	GGTTTTTGAGGGTGAGGGTGAGGGTGAGGGTGAGGGT	telomere content
*telom*-Rv	TCCCGAACTATCCCTATCCCTATCCCTATCCCTATCCCTA	telomere content
*rps11*-Fw	ACAGAAATGCCCCTTCACTG	gene expresión telomere content
*rps11*-Rv	GCCTCTTCTCAAAACGGTTG	gene expresión telomere content
