# Supplementary figures and images for: Synergizing Virtual Screening and Zebrafish Models to Identify Resveratrol-Derived Antiaging Polyphenols
Source: Pharmaceuticals (Basel). 2025 Oct 28;18(11):1630. doi: 10.3390/ph18111630 (PMC12655600; doi:10.3390/ph18111630)

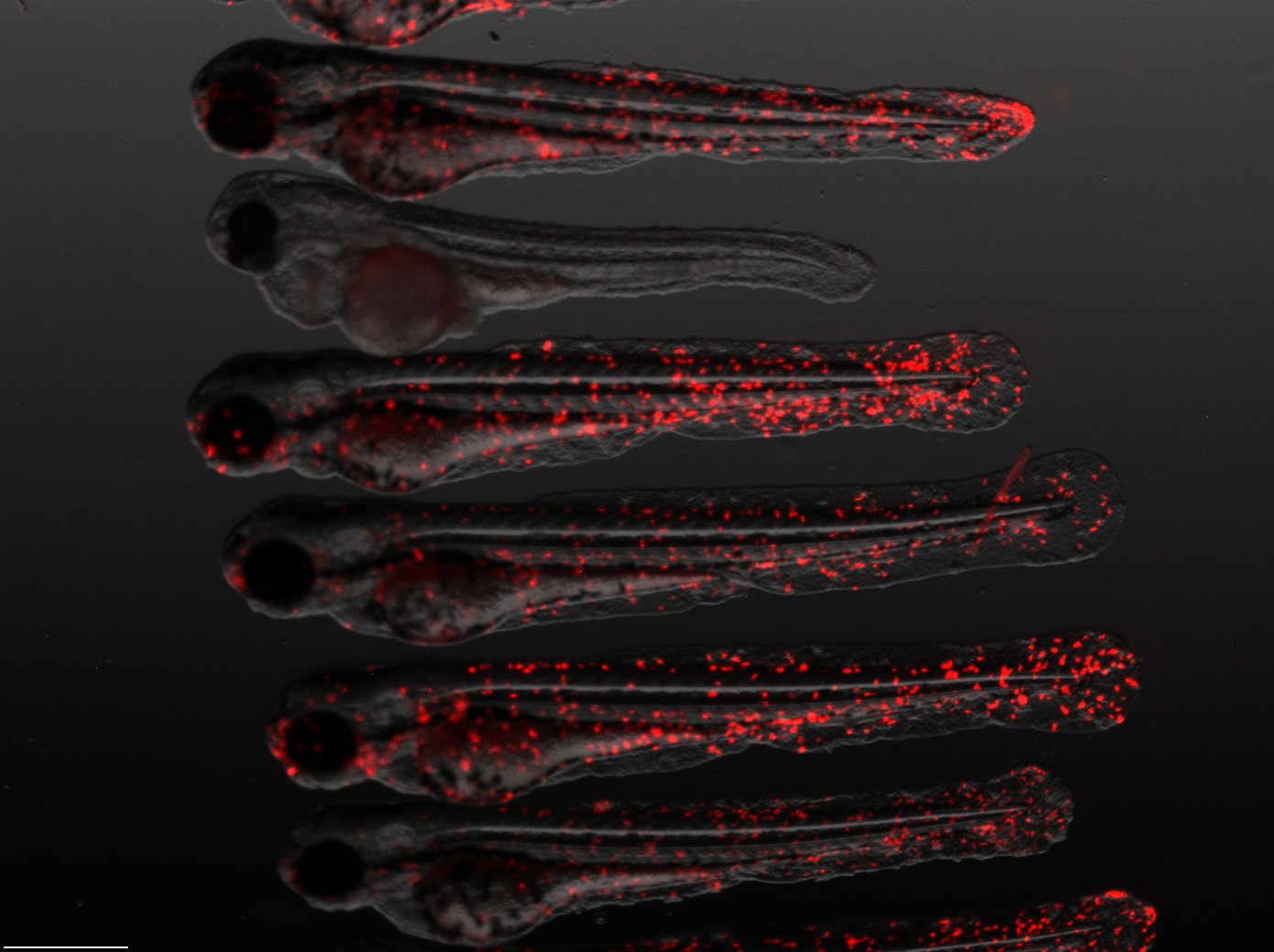

Supplement: Supplementary file 1 [file pharmaceuticals-18-01630-s001.zip › Images Fig. 5A/hai -- c-_Image028.tif]

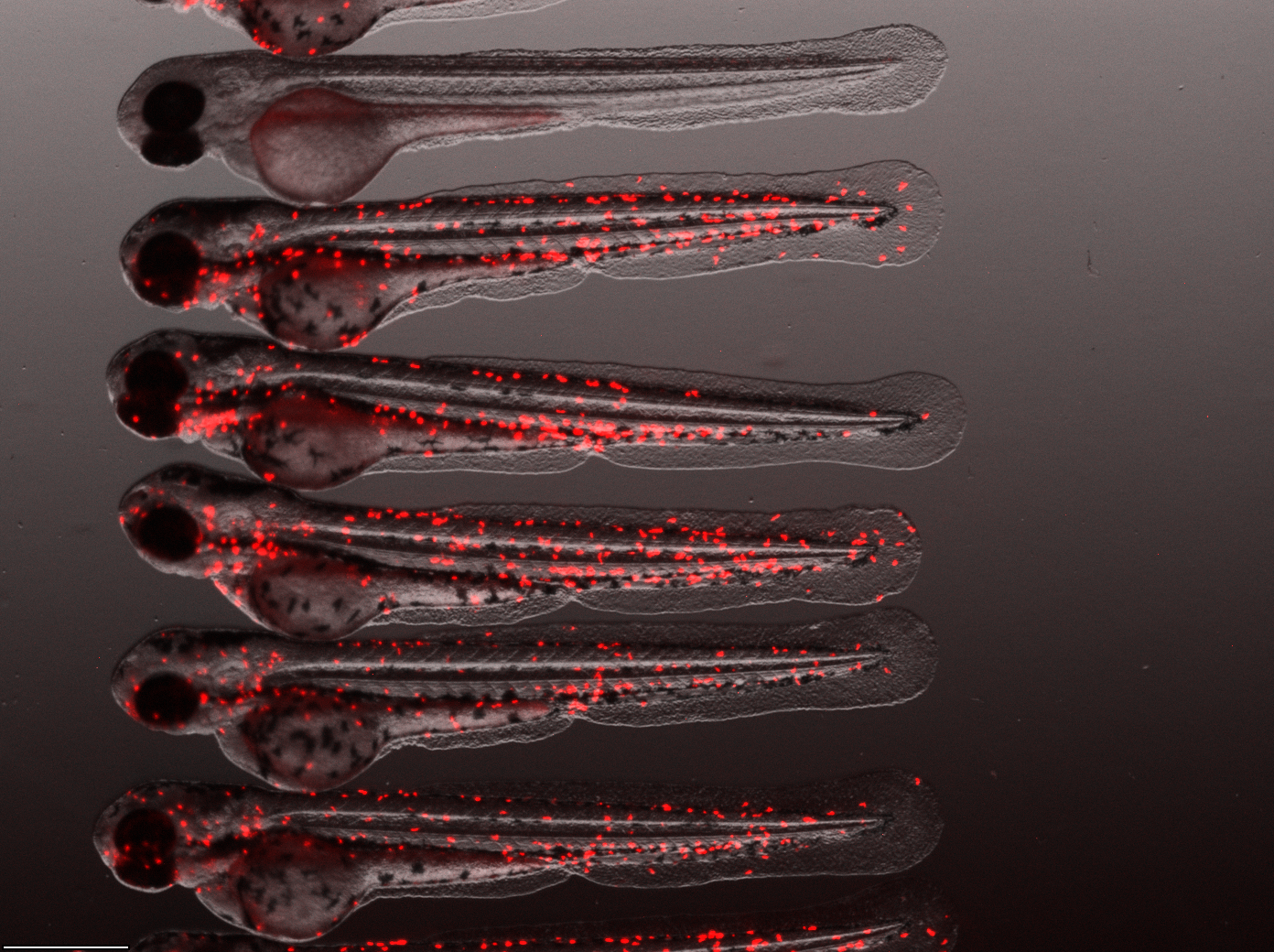

Supplement: Supplementary file 1 [file pharmaceuticals-18-01630-s001.zip › Images Fig. 5A/hai -- genestein_Image046.tif]

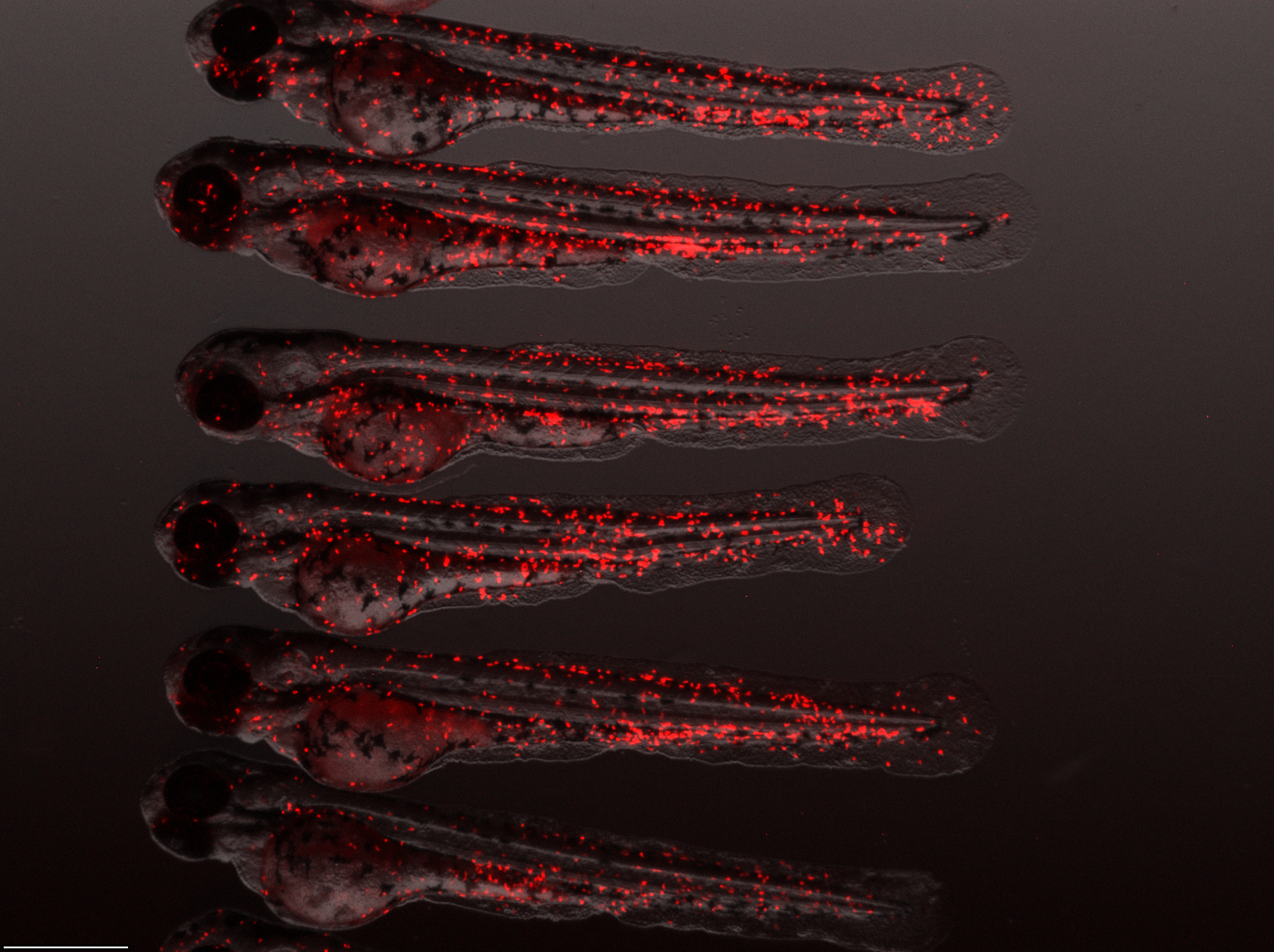

Supplement: Supplementary file 1 [file pharmaceuticals-18-01630-s001.zip › Images Fig. 5A/hai -- hespe_Image022.tif]

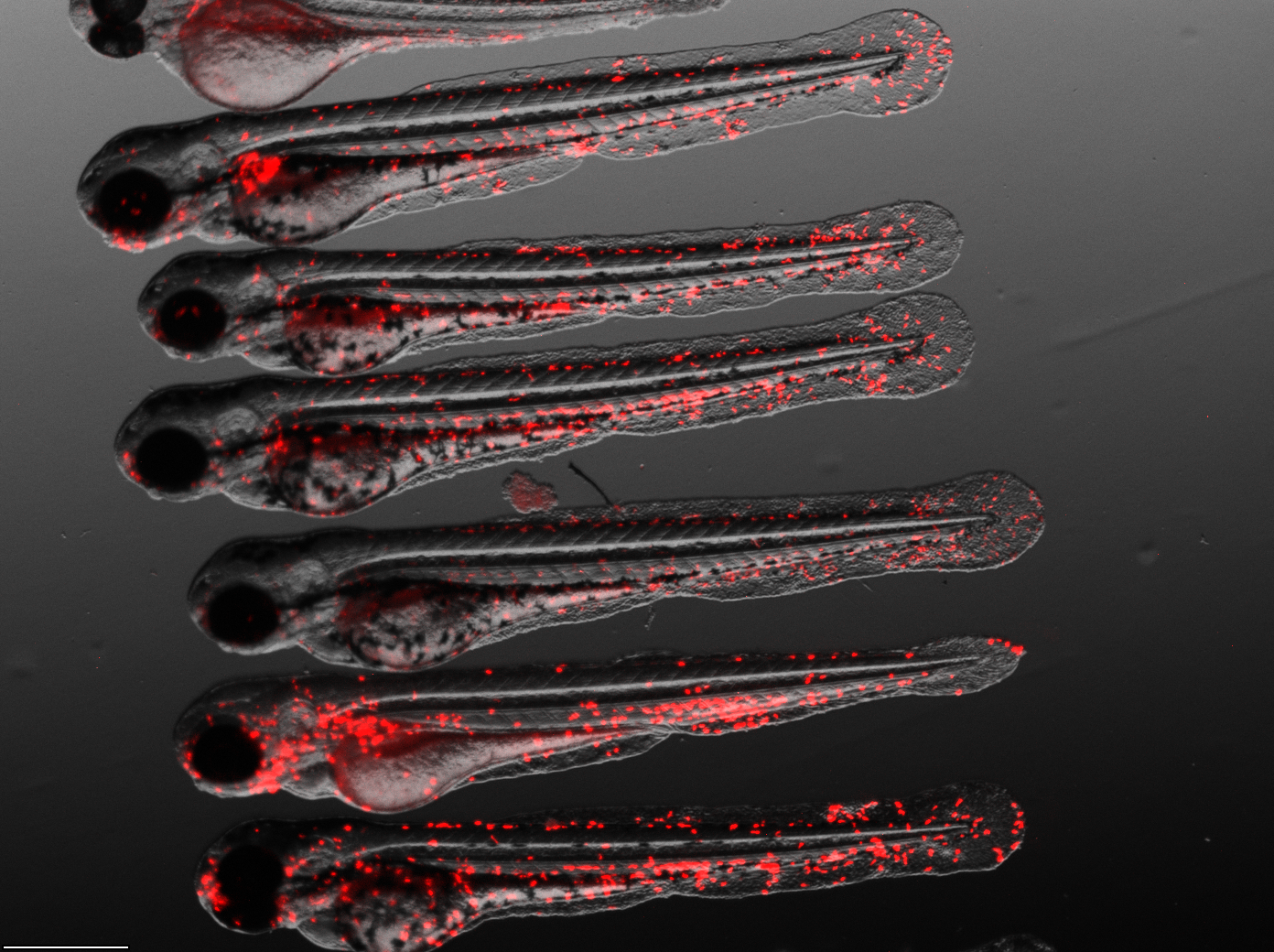

Supplement: Supplementary file 1 [file pharmaceuticals-18-01630-s001.zip › Images Fig. 5A/hai -- liquircetin_Image061.tif]

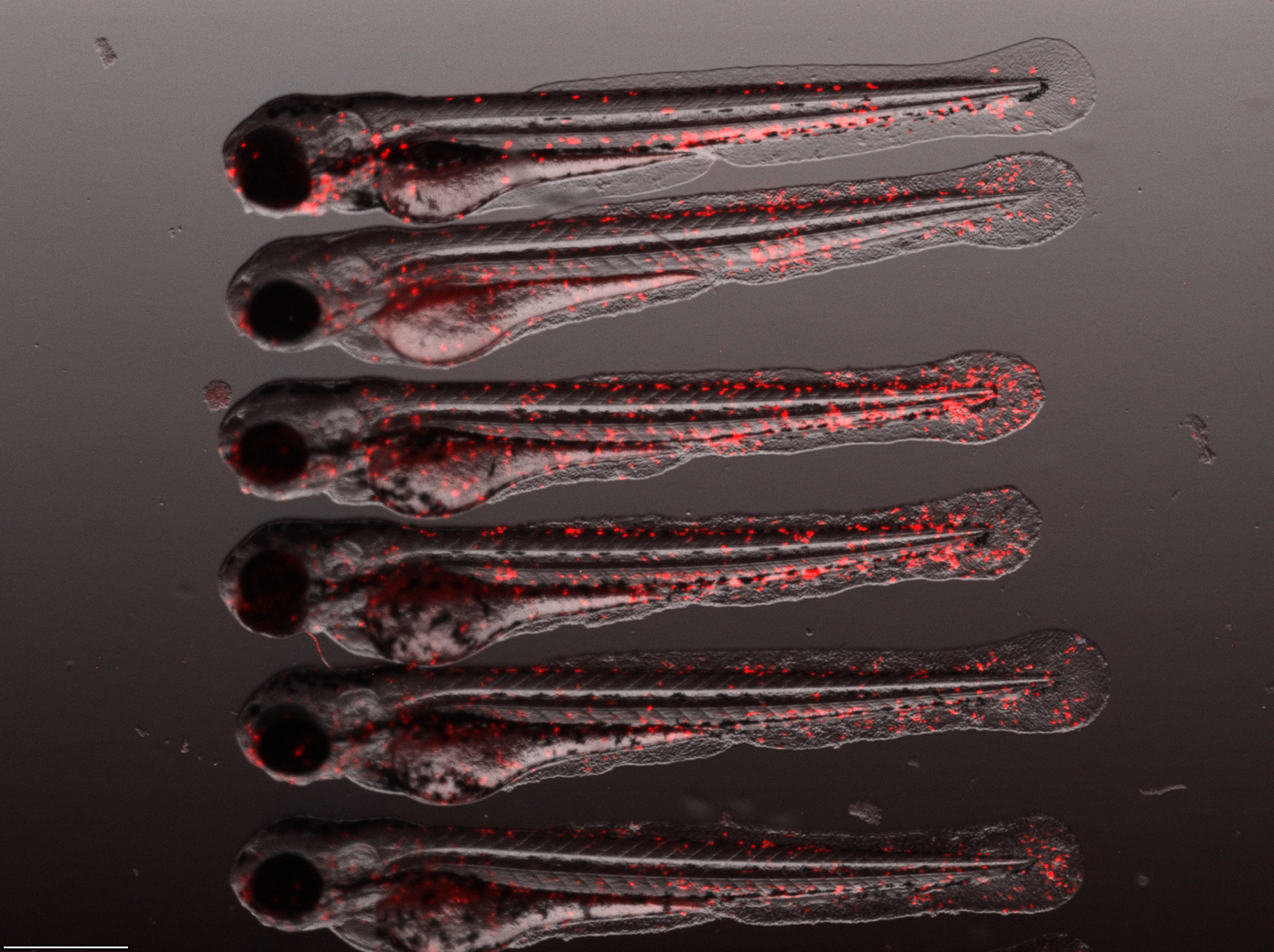

Supplement: Supplementary file 1 [file pharmaceuticals-18-01630-s001.zip › Images Fig. 5A/hai -- narigenin_Image064.tif]

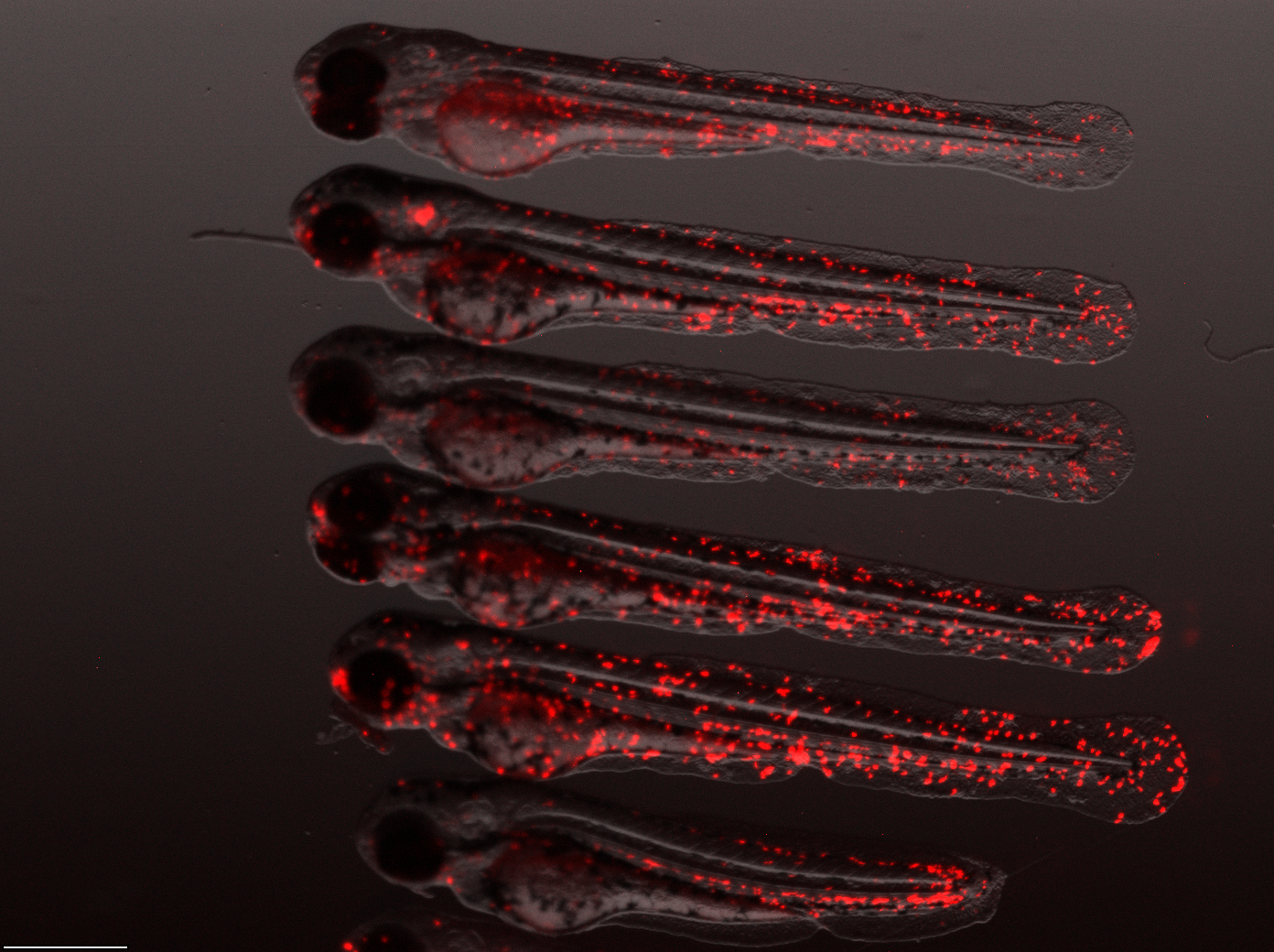

Supplement: Supplementary file 1 [file pharmaceuticals-18-01630-s001.zip › Images Fig. 5A/hai -- quercetin_Image030.tif]

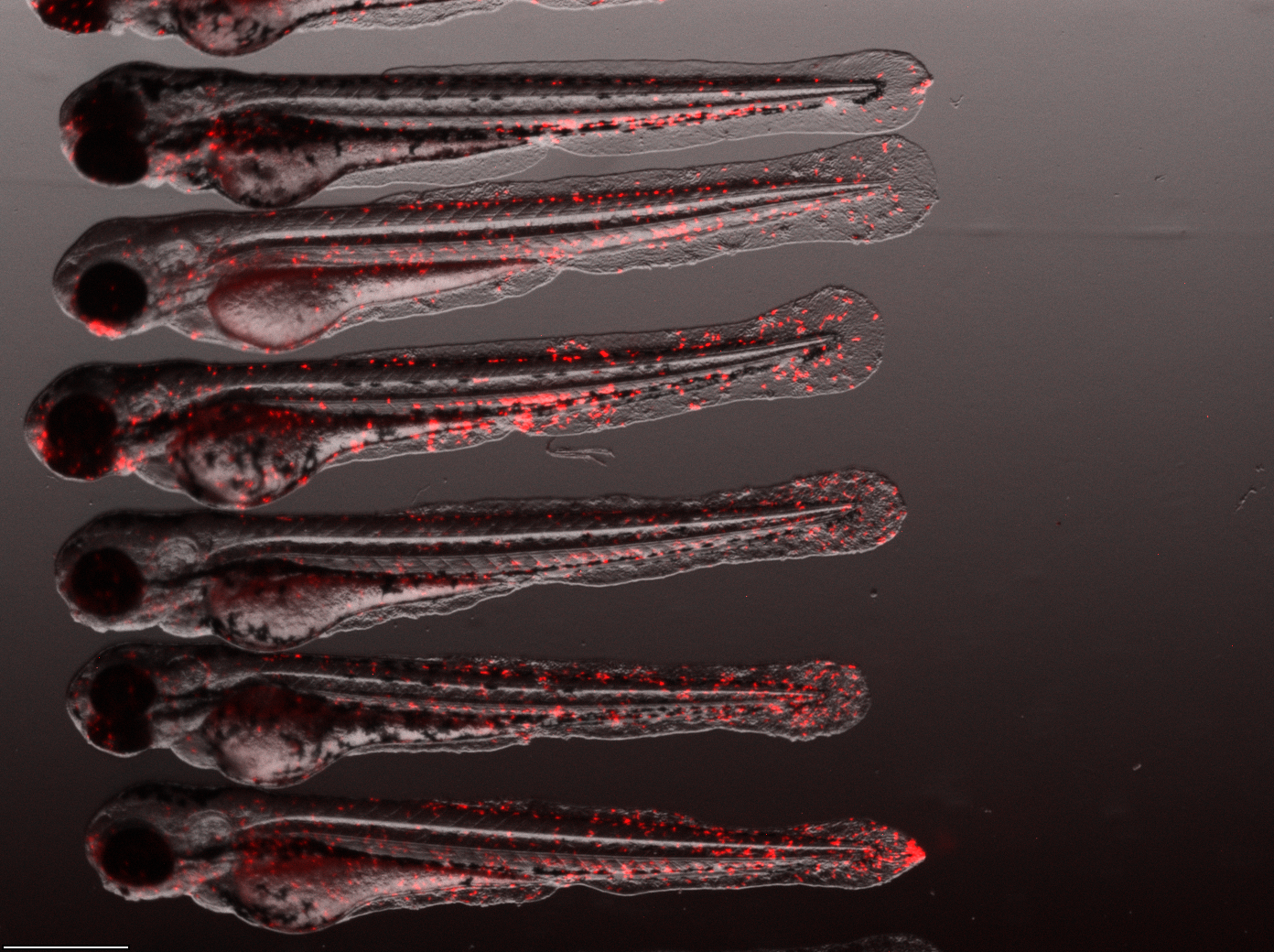

Supplement: Supplementary file 1 [file pharmaceuticals-18-01630-s001.zip › Images Fig. 5A/hai -- sakuretin_Image042.tif]

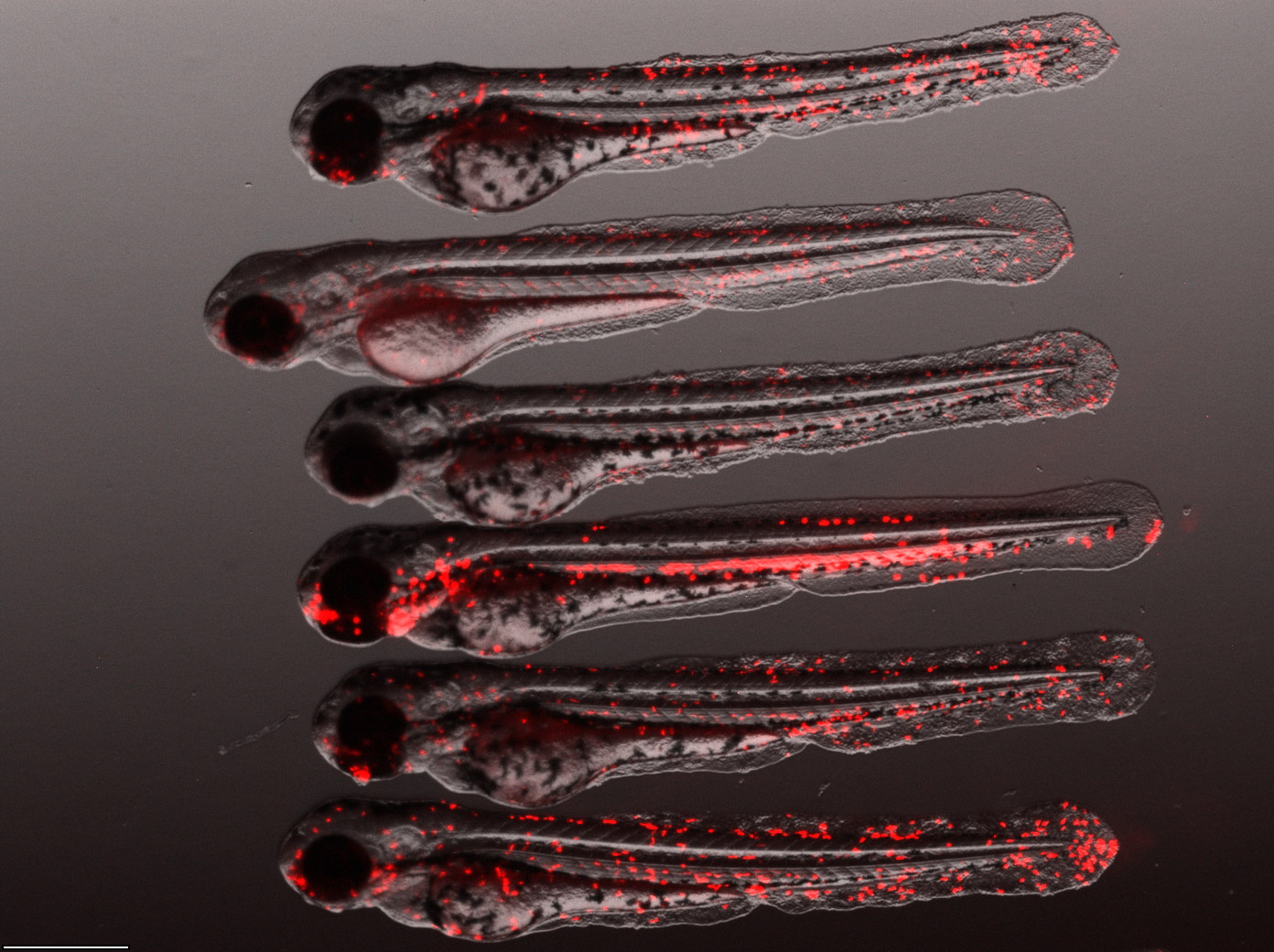

Supplement: Supplementary file 1 [file pharmaceuticals-18-01630-s001.zip › Images Fig. 5A/hai -- tricetin_Image034.tif]

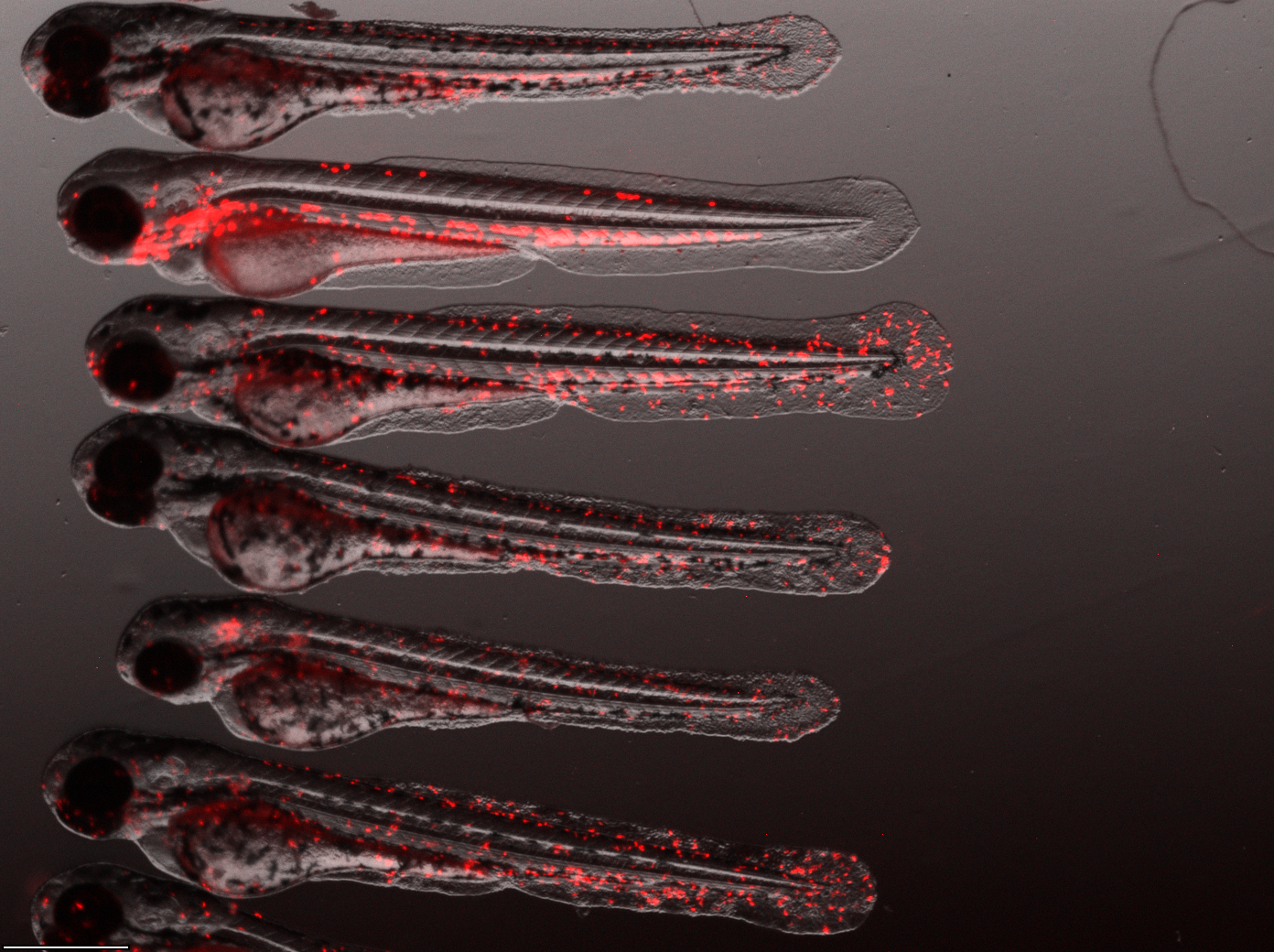

Supplement: Supplementary file 1 [file pharmaceuticals-18-01630-s001.zip › Images Fig. 5A/hai-- apigenina_Image058.tif]

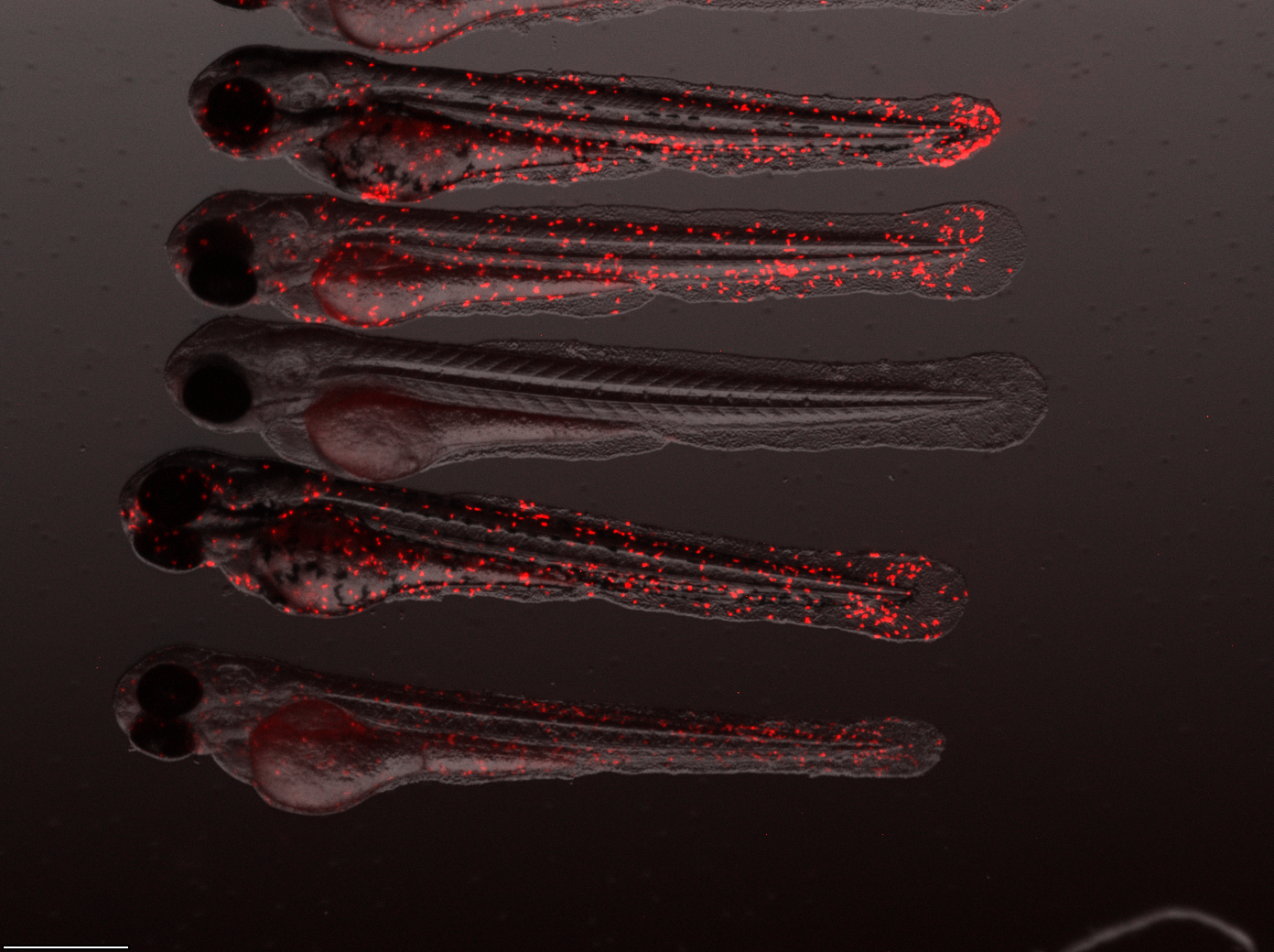

Supplement: Supplementary file 1 [file pharmaceuticals-18-01630-s001.zip › Images Fig. 5A/hai-- resveratrol_Image005.tif]

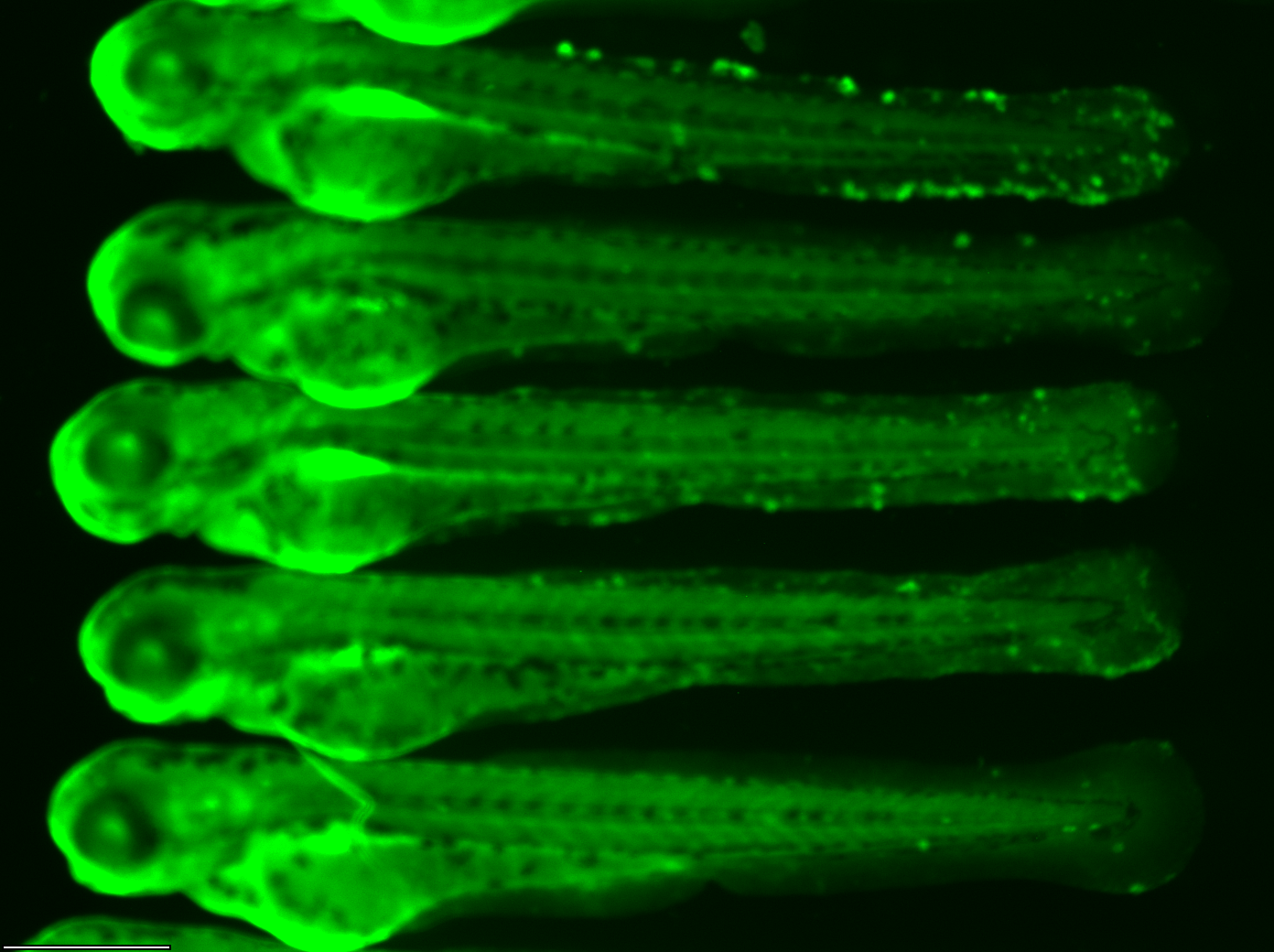

Supplement: Supplementary file 1 [file pharmaceuticals-18-01630-s001.zip › Images Fig. 5D/apigenin_larvae#2.tif]

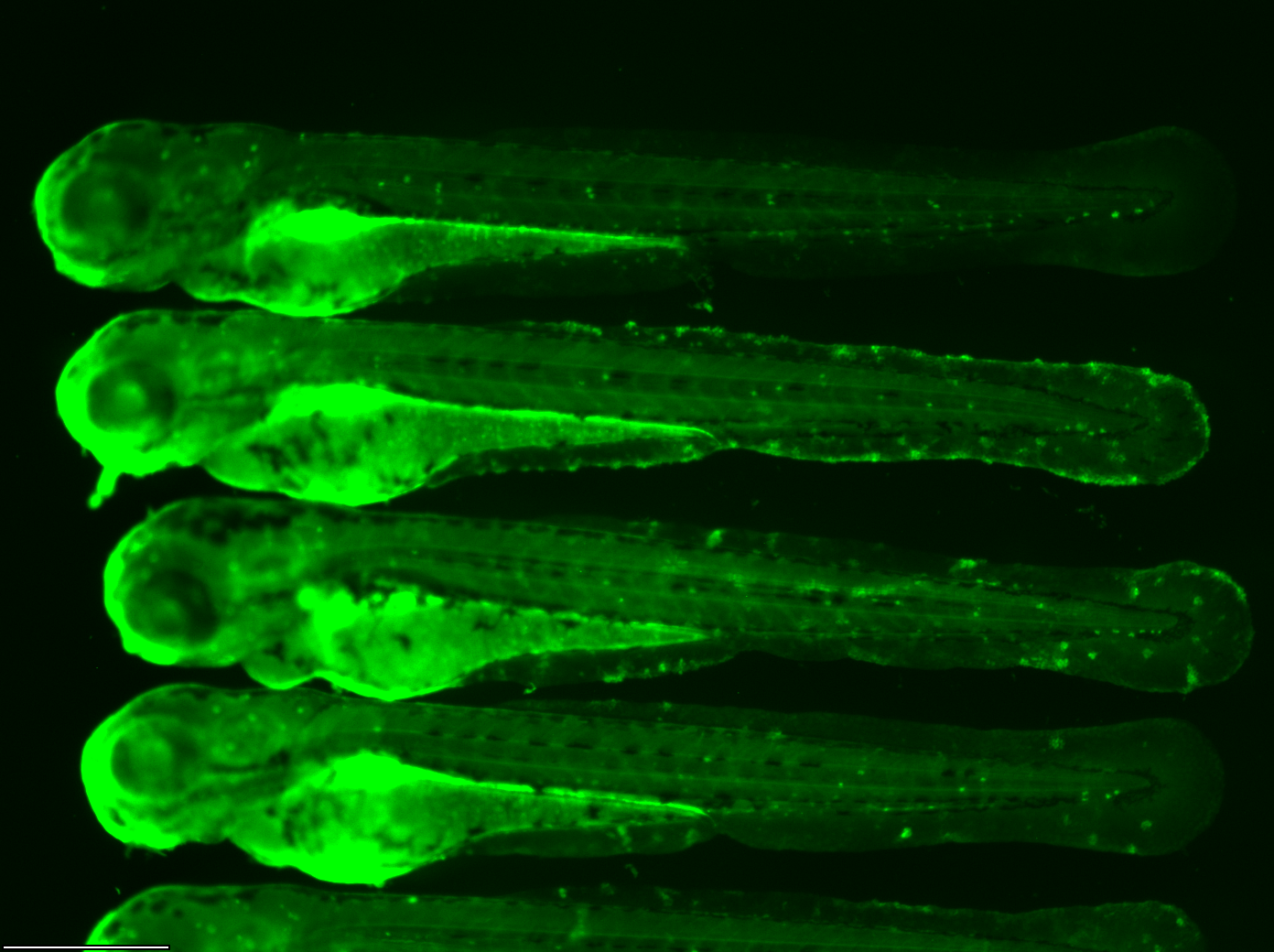

Supplement: Supplementary file 1 [file pharmaceuticals-18-01630-s001.zip › Images Fig. 5D/DMSO_larvae#2.tif]

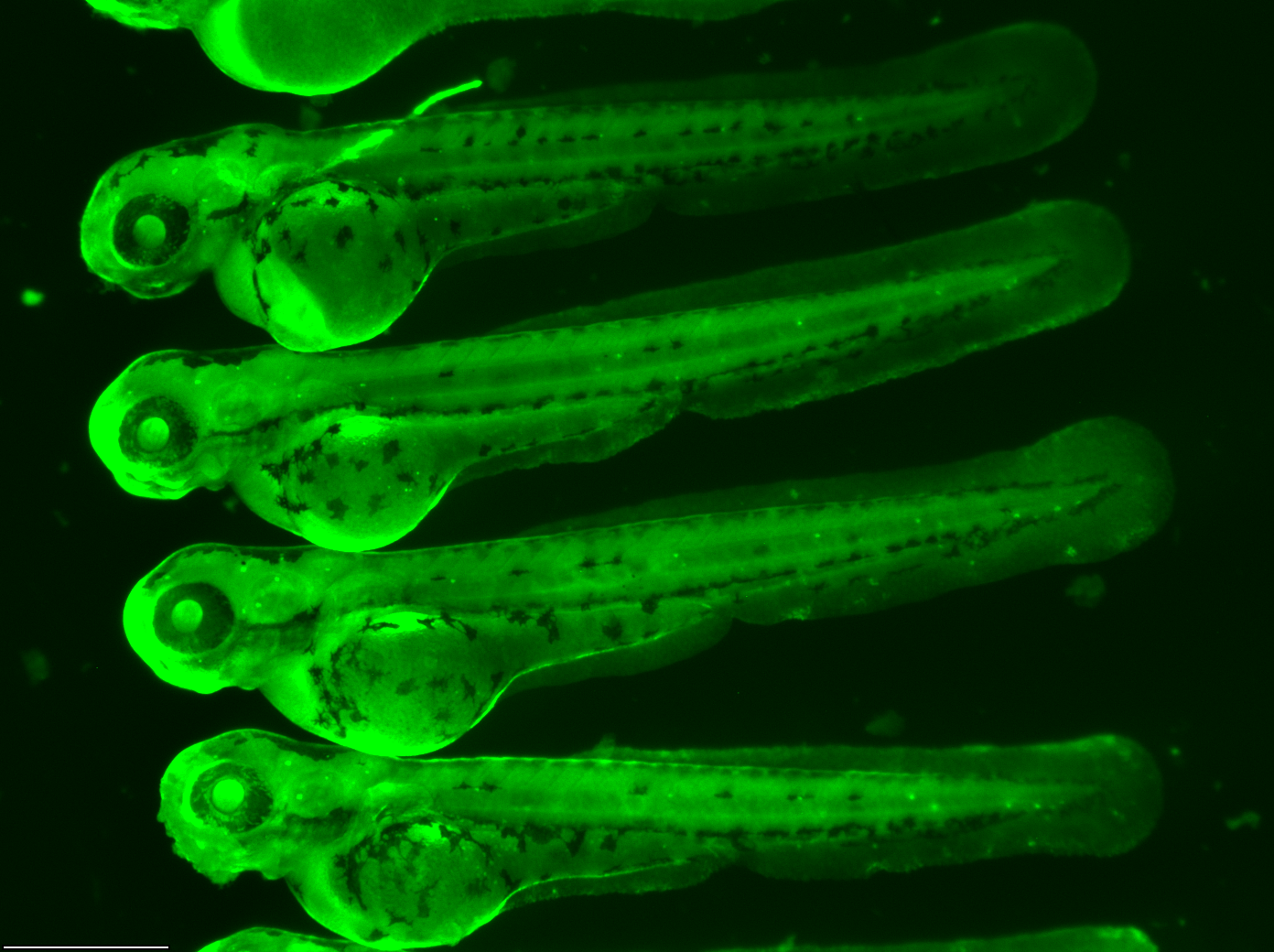

Supplement: Supplementary file 1 [file pharmaceuticals-18-01630-s001.zip › Images Fig. 5D/genestein_larvae#1.tif]

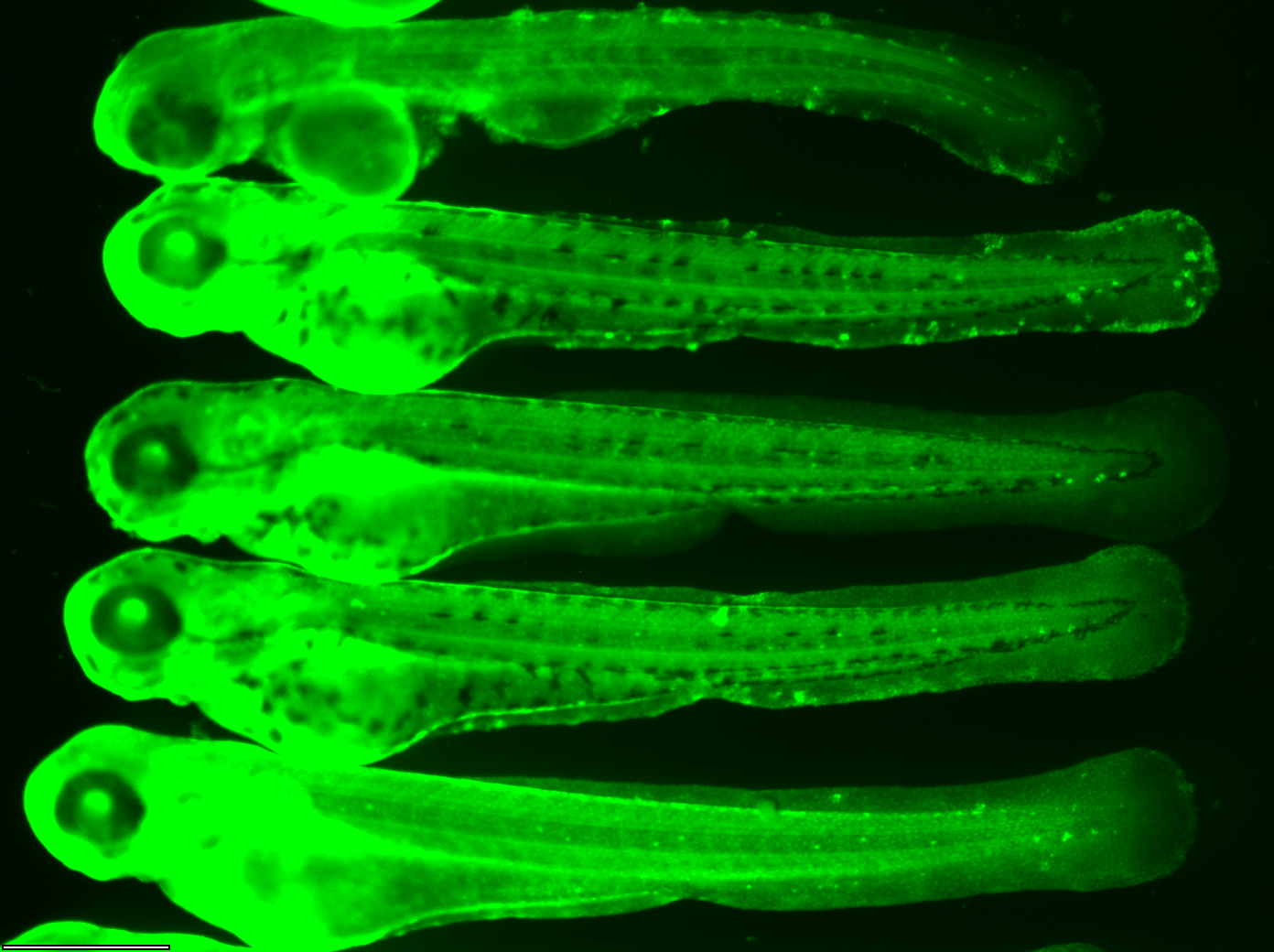

Supplement: Supplementary file 1 [file pharmaceuticals-18-01630-s001.zip › Images Fig. 5D/hesperetin_larvae#3.tif]

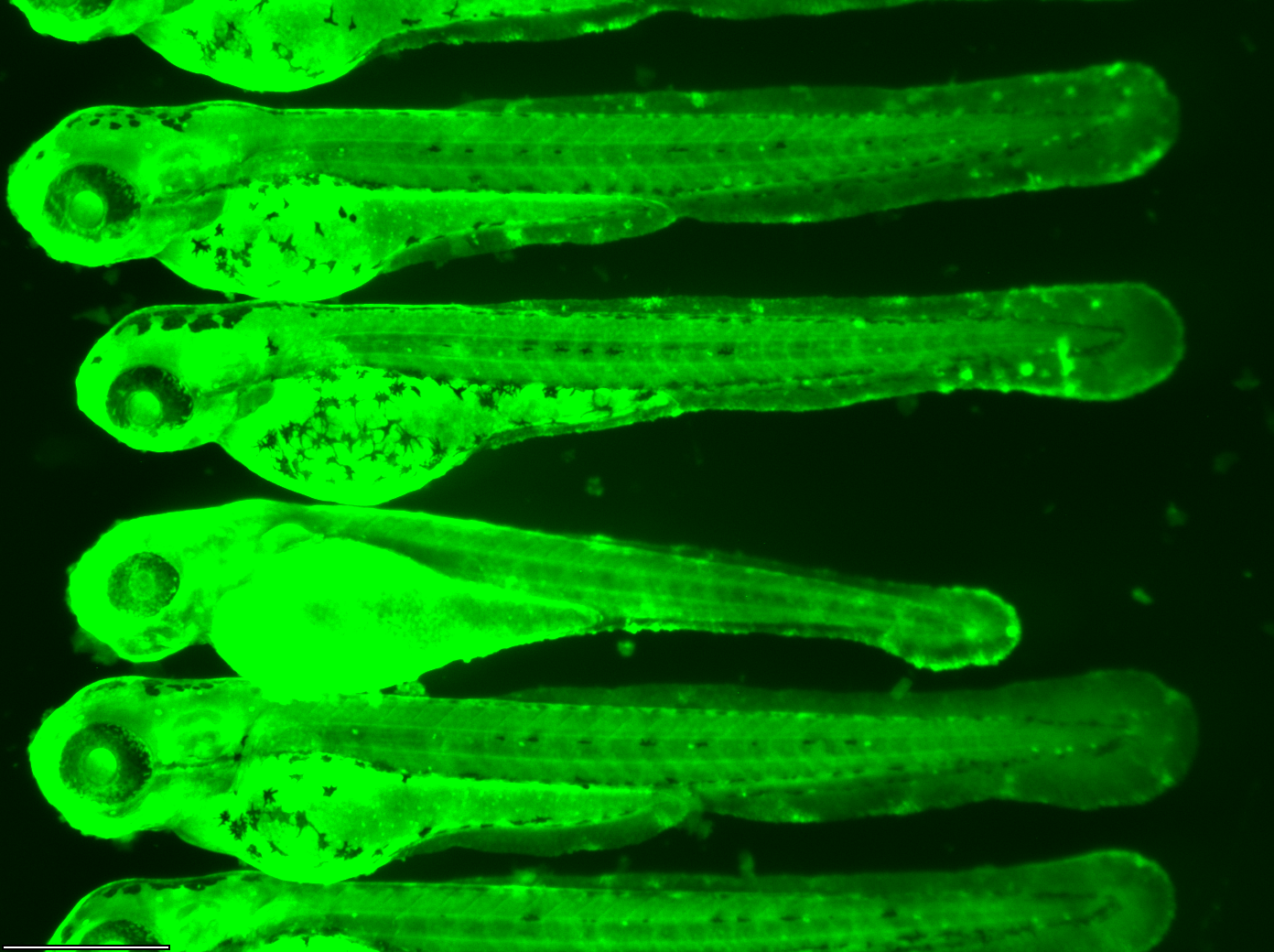

Supplement: Supplementary file 1 [file pharmaceuticals-18-01630-s001.zip › Images Fig. 5D/liquiritigenin_larvae#4.tif]

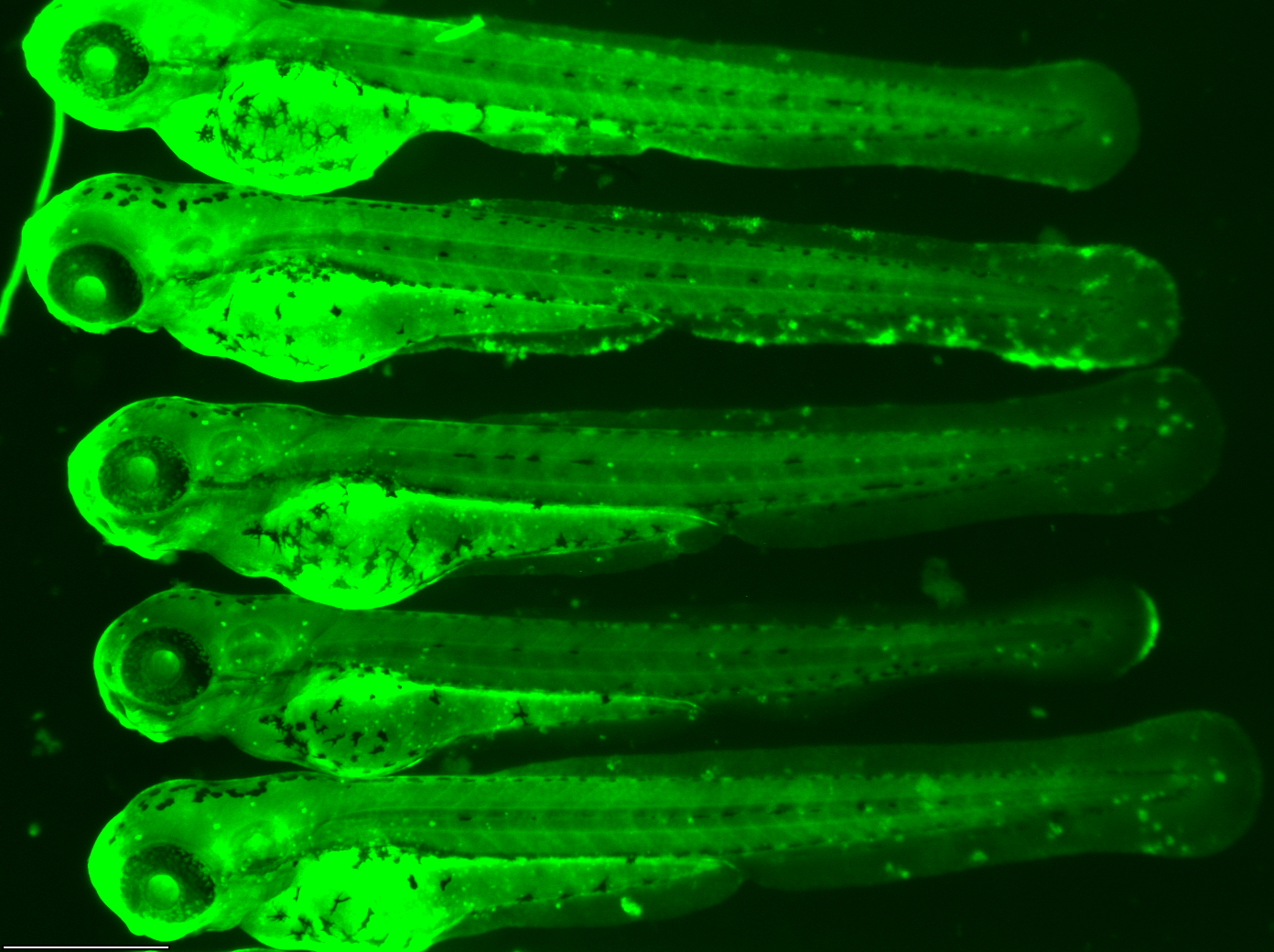

Supplement: Supplementary file 1 [file pharmaceuticals-18-01630-s001.zip › Images Fig. 5D/naringenin_larvae#5.tif]

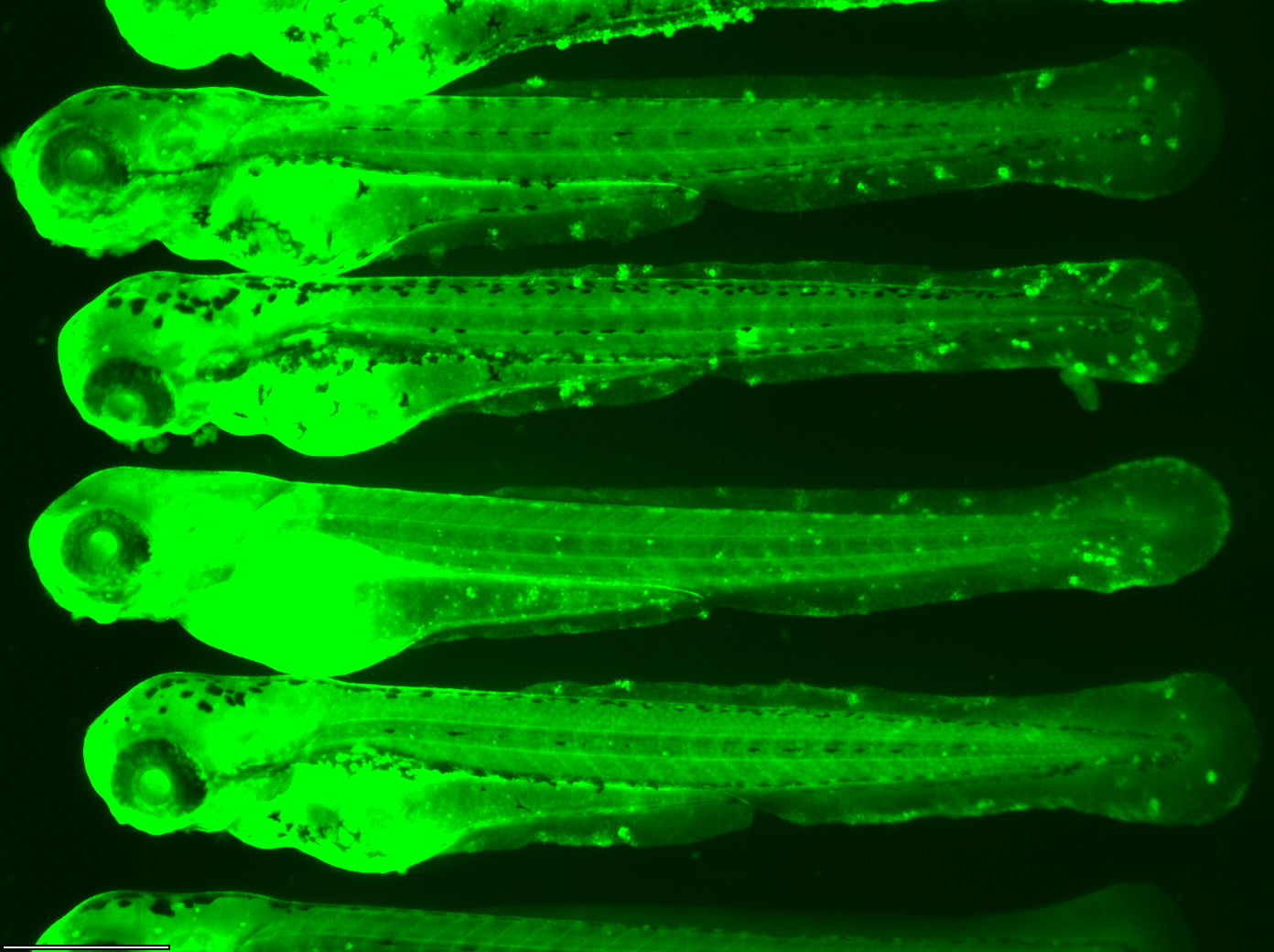

Supplement: Supplementary file 1 [file pharmaceuticals-18-01630-s001.zip › Images Fig. 5D/quercetin_larvae#1.tif]

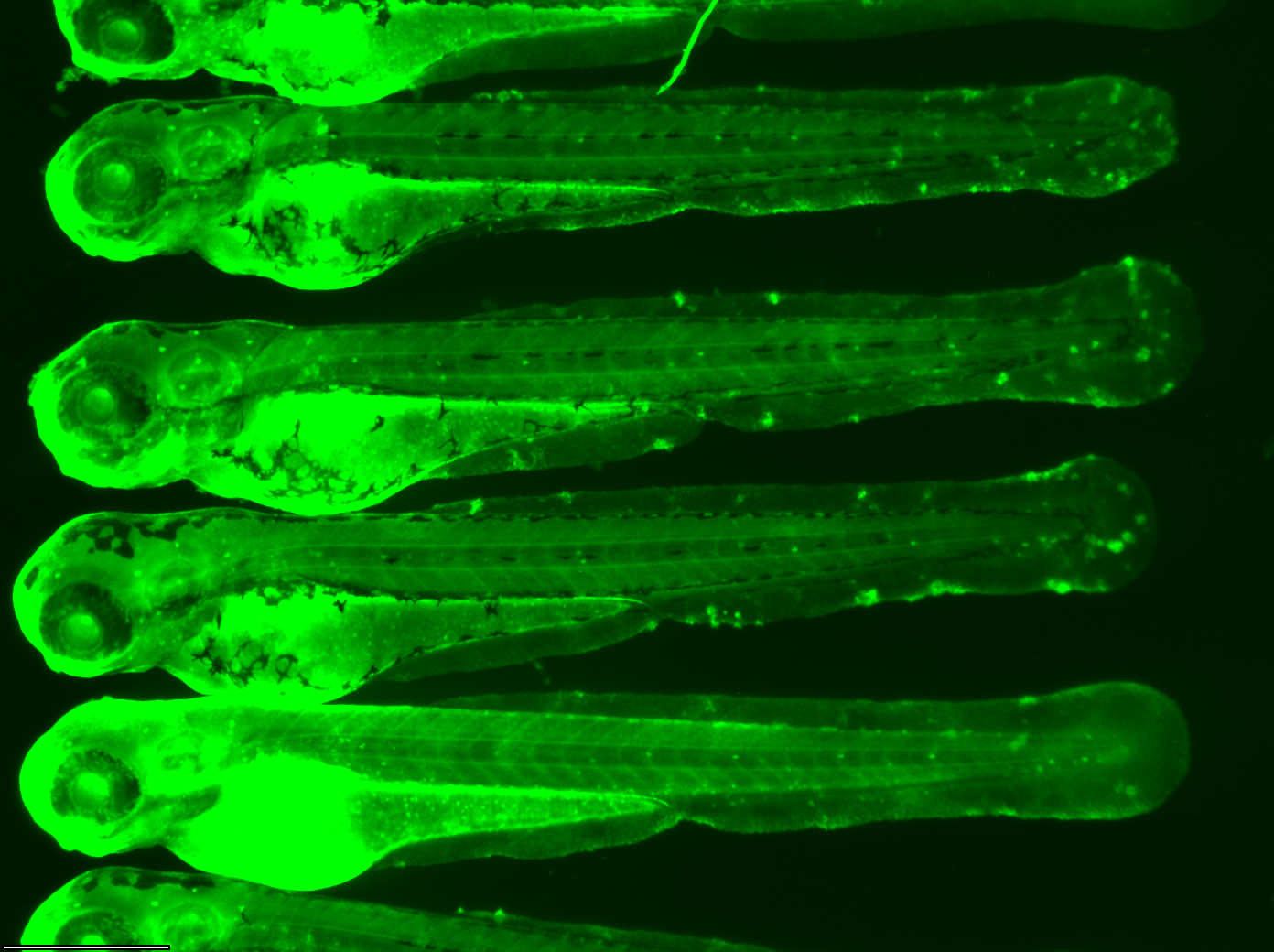

Supplement: Supplementary file 1 [file pharmaceuticals-18-01630-s001.zip › Images Fig. 5D/resveratrol_larvae#2.tif]

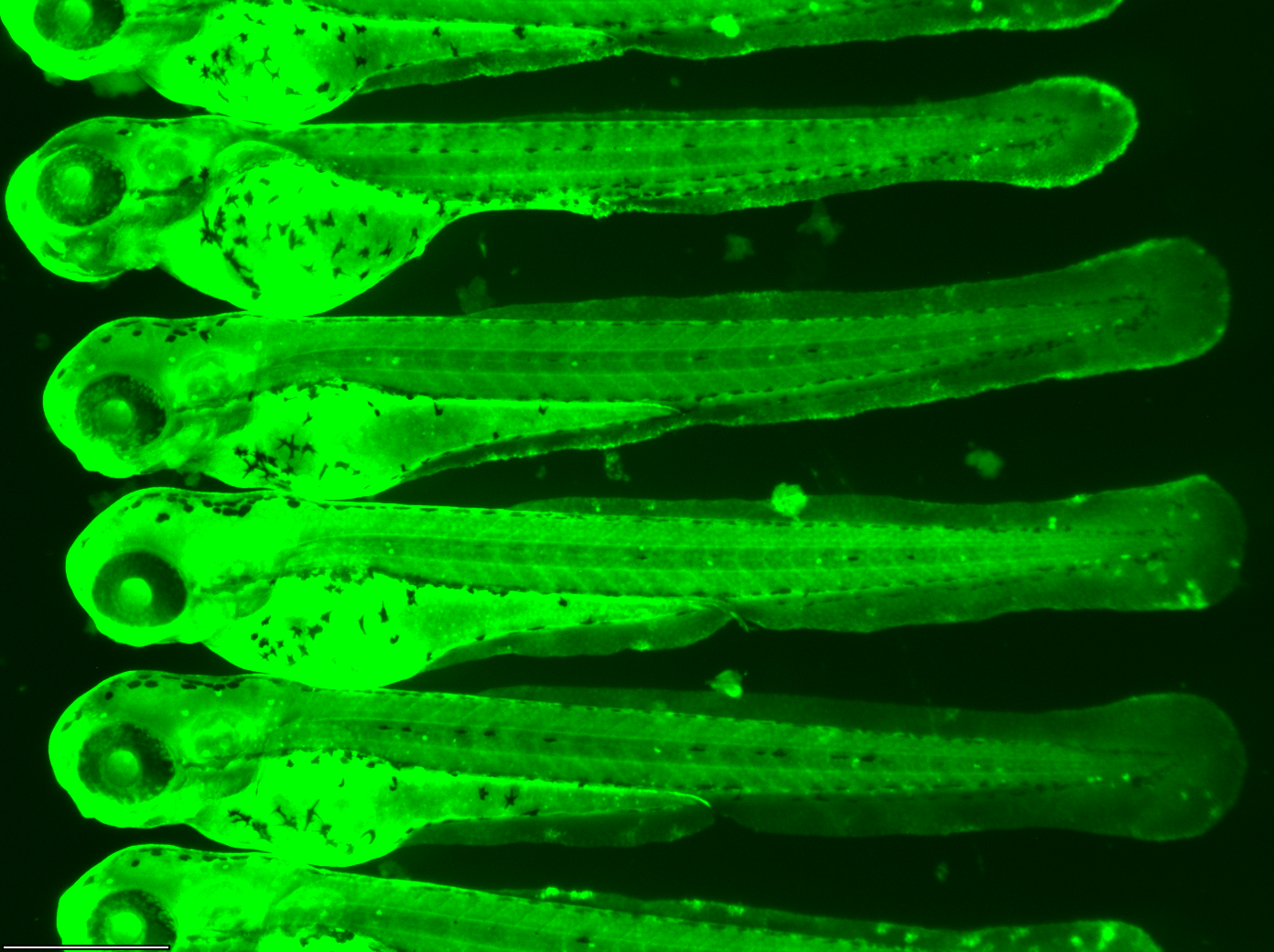

Supplement: Supplementary file 1 [file pharmaceuticals-18-01630-s001.zip › Images Fig. 5D/sakuranetin_larvae#4.tif]

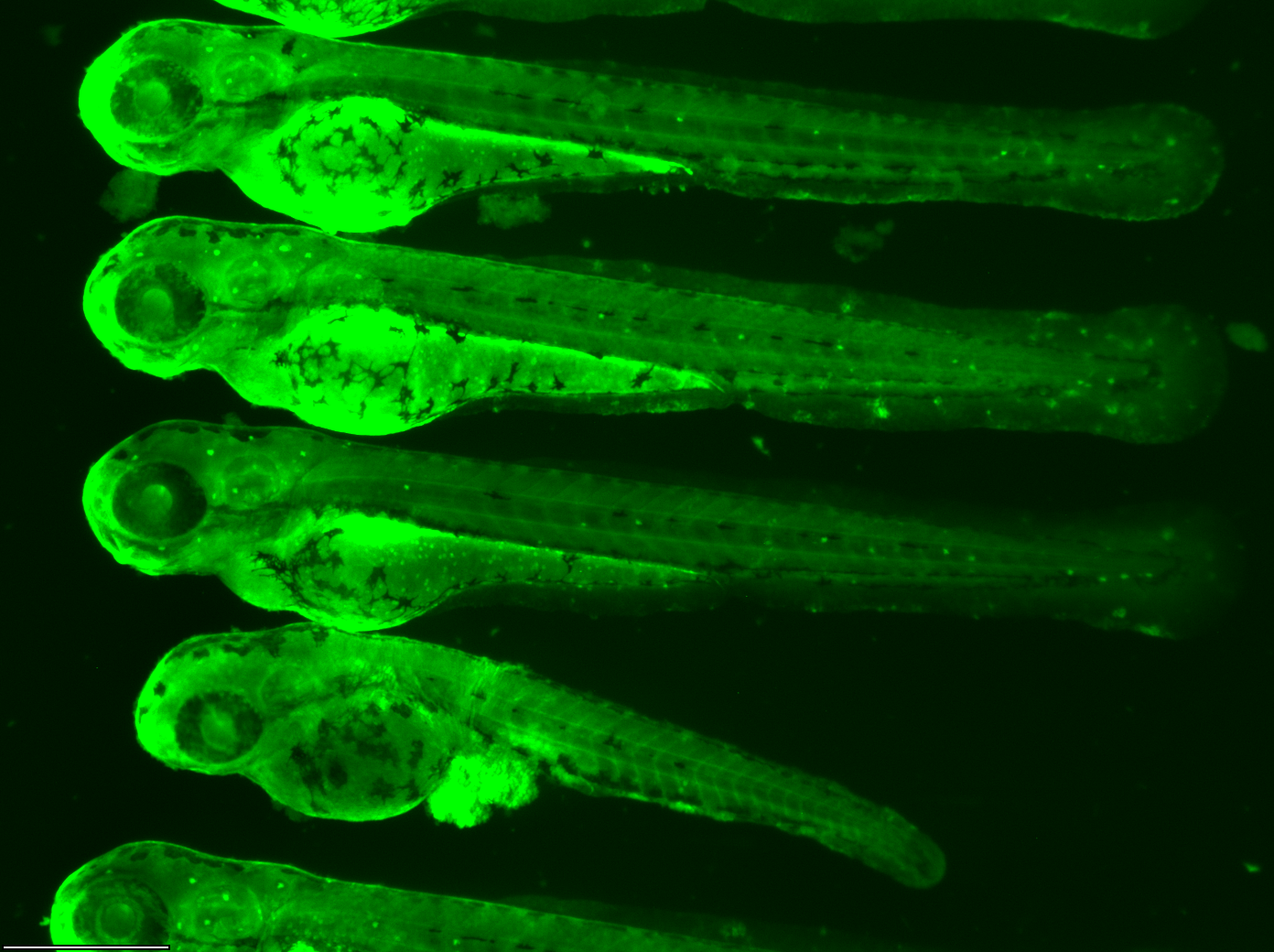

Supplement: Supplementary file 1 [file pharmaceuticals-18-01630-s001.zip › Images Fig. 5D/tricetin_larvae#2.tif]

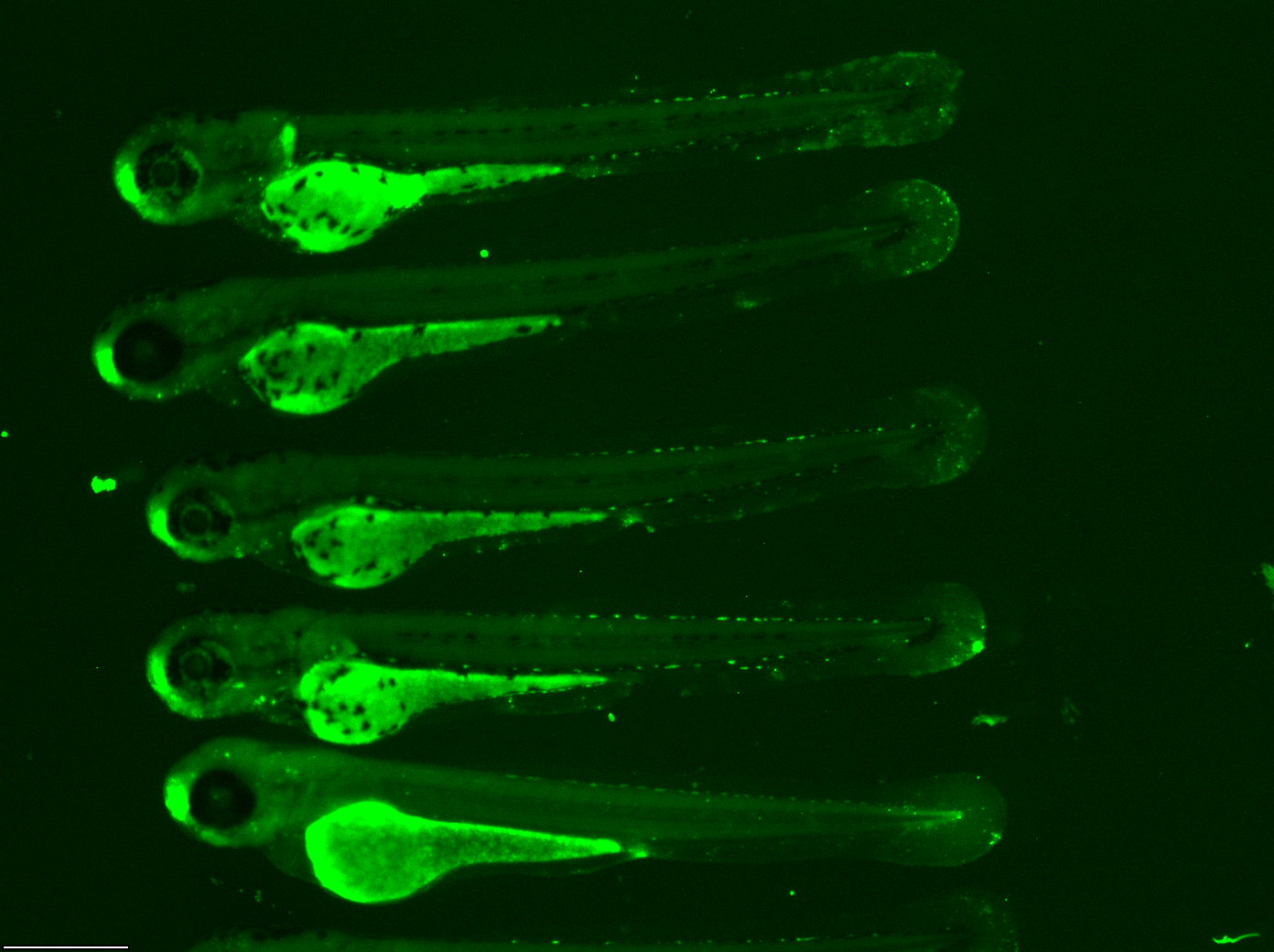

Supplement: Supplementary file 1 [file pharmaceuticals-18-01630-s001.zip › Images Fig. 5F/apigenin_larvae#3.tif]

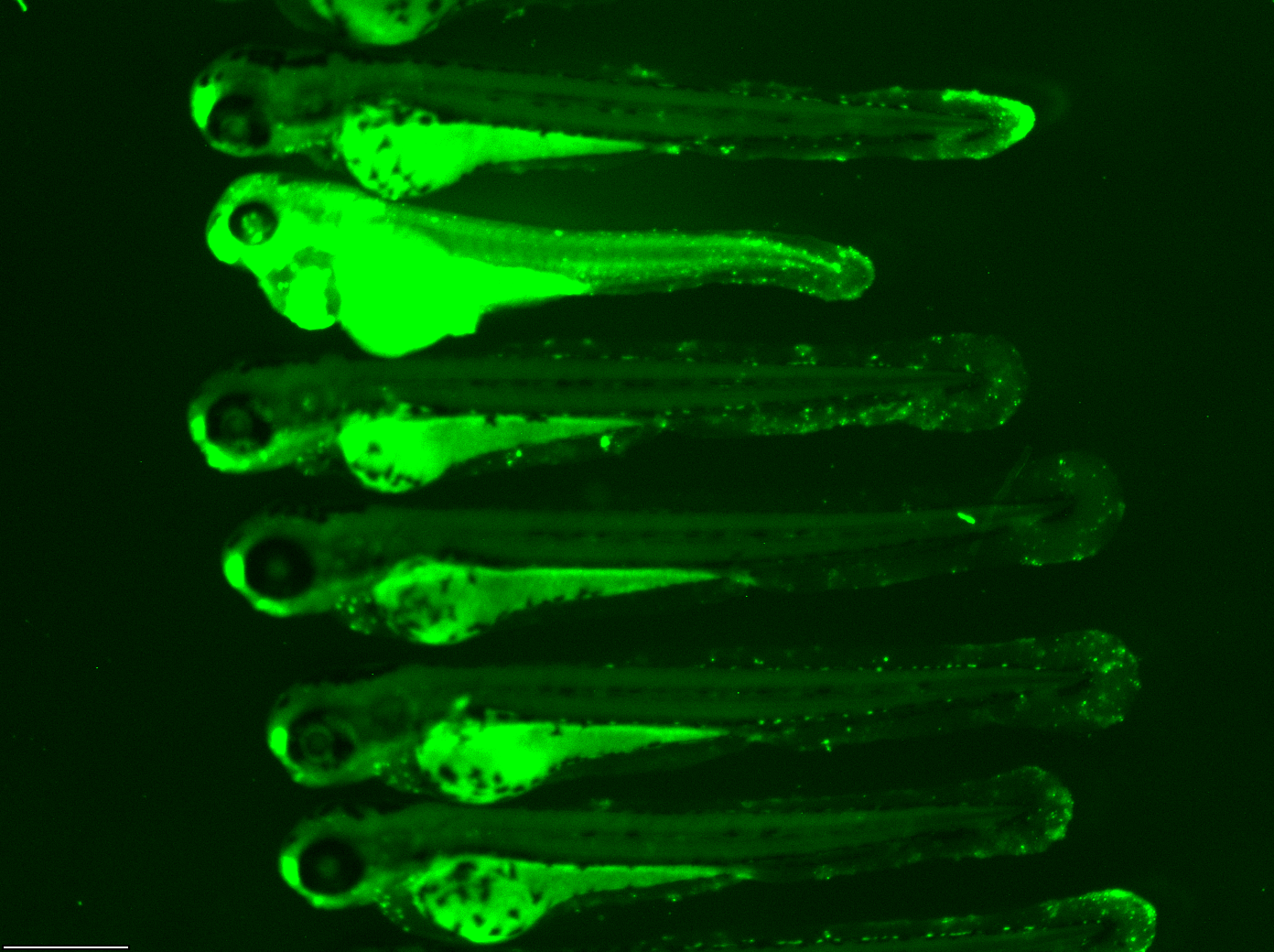

Supplement: Supplementary file 1 [file pharmaceuticals-18-01630-s001.zip › Images Fig. 5F/DMSO_larvae#3.tif]

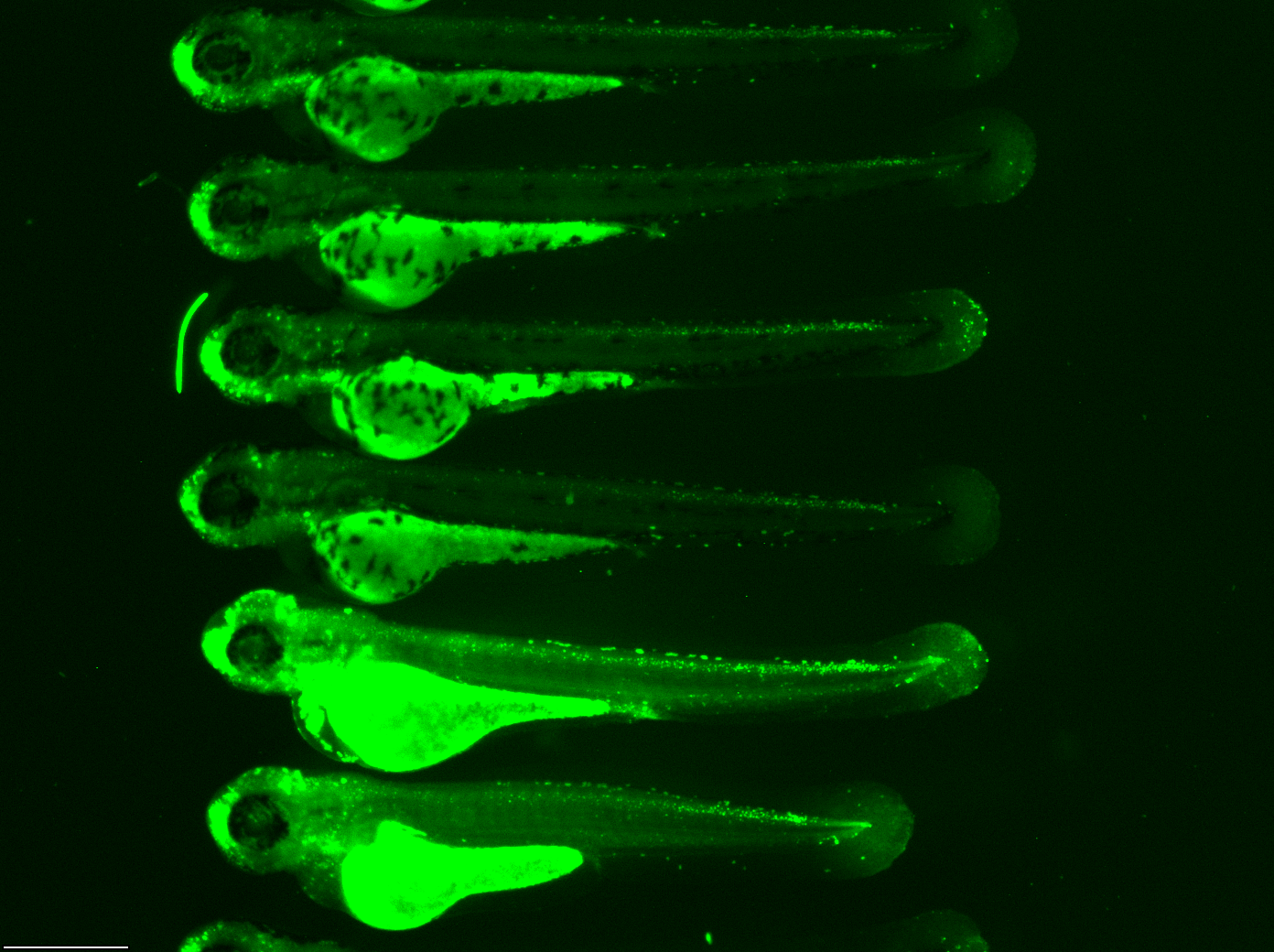

Supplement: Supplementary file 1 [file pharmaceuticals-18-01630-s001.zip › Images Fig. 5F/genestein_larvae#2.tif]

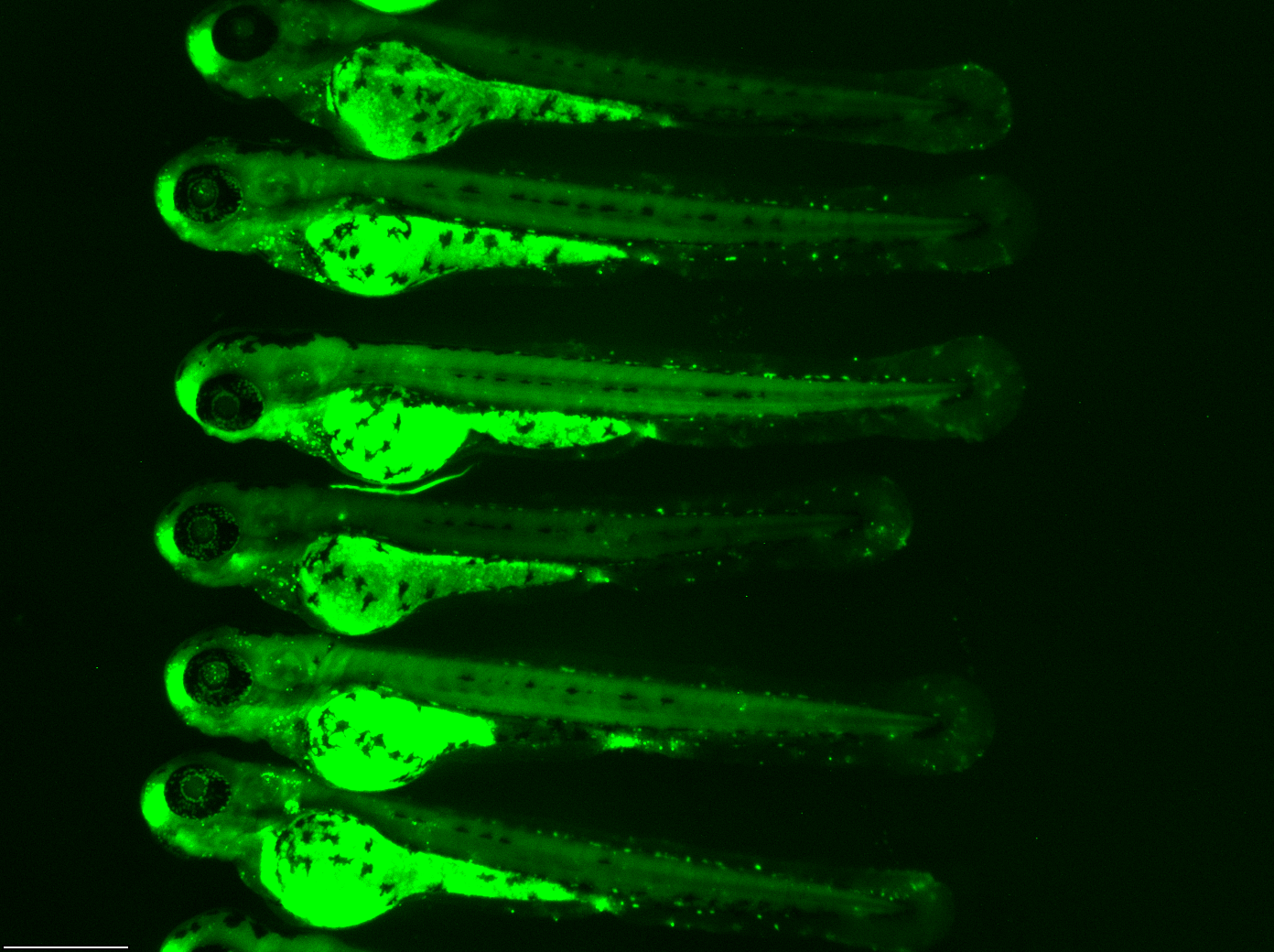

Supplement: Supplementary file 1 [file pharmaceuticals-18-01630-s001.zip › Images Fig. 5F/hesperetin_larvae#2.tif]

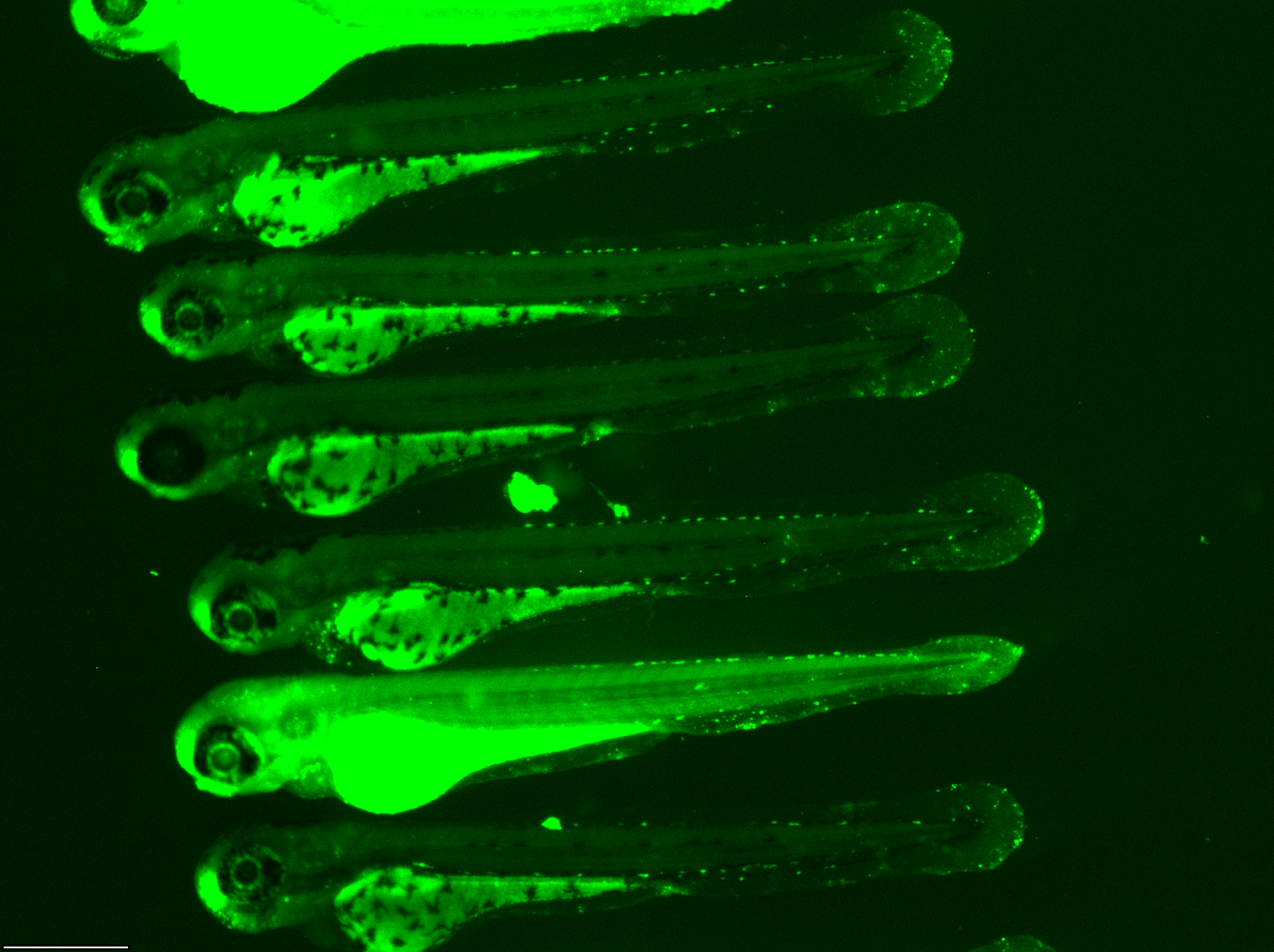

Supplement: Supplementary file 1 [file pharmaceuticals-18-01630-s001.zip › Images Fig. 5F/liquiritigenin_larvae#4.tif]

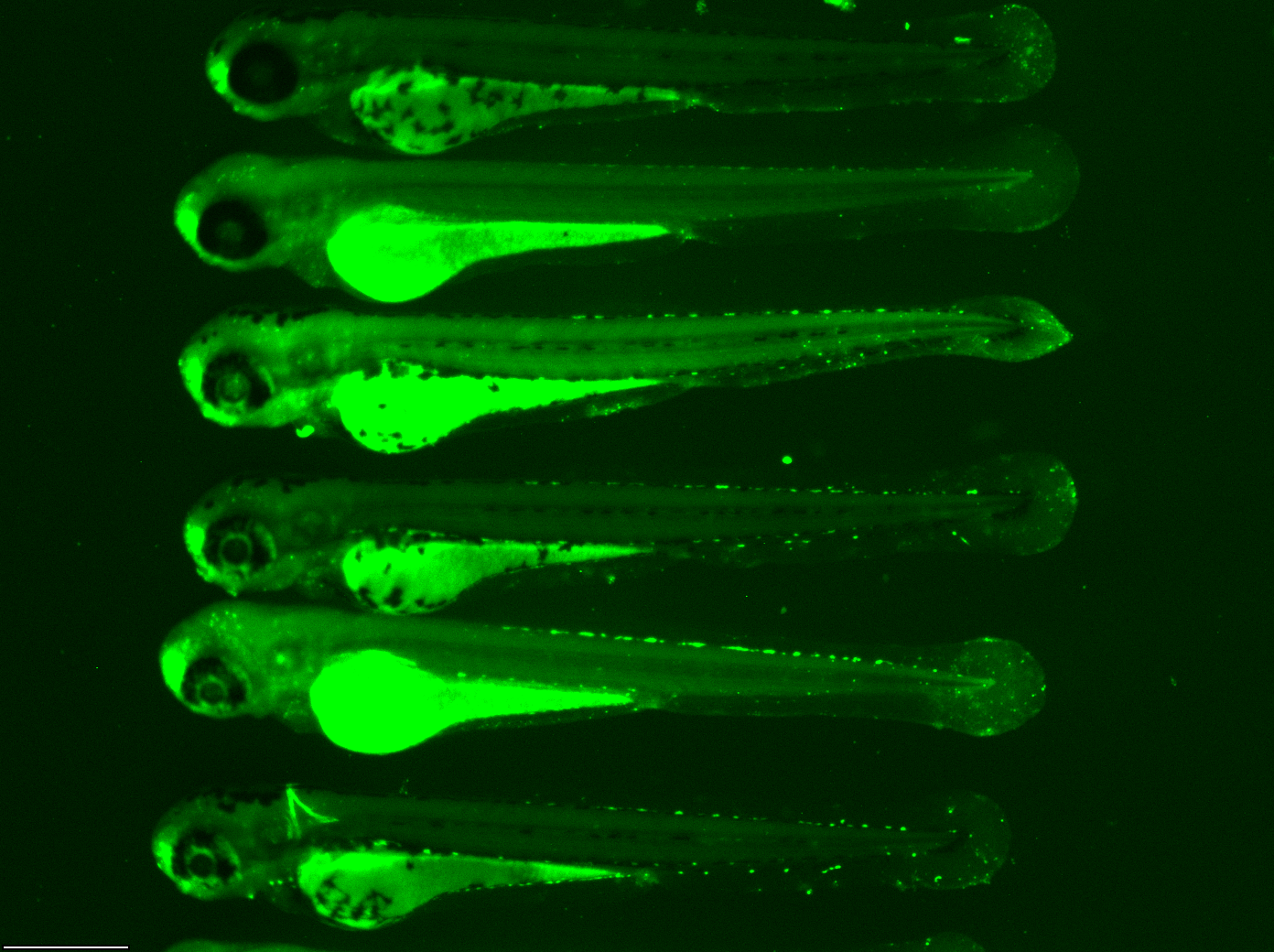

Supplement: Supplementary file 1 [file pharmaceuticals-18-01630-s001.zip › Images Fig. 5F/naringenin_larvae#6.tif]

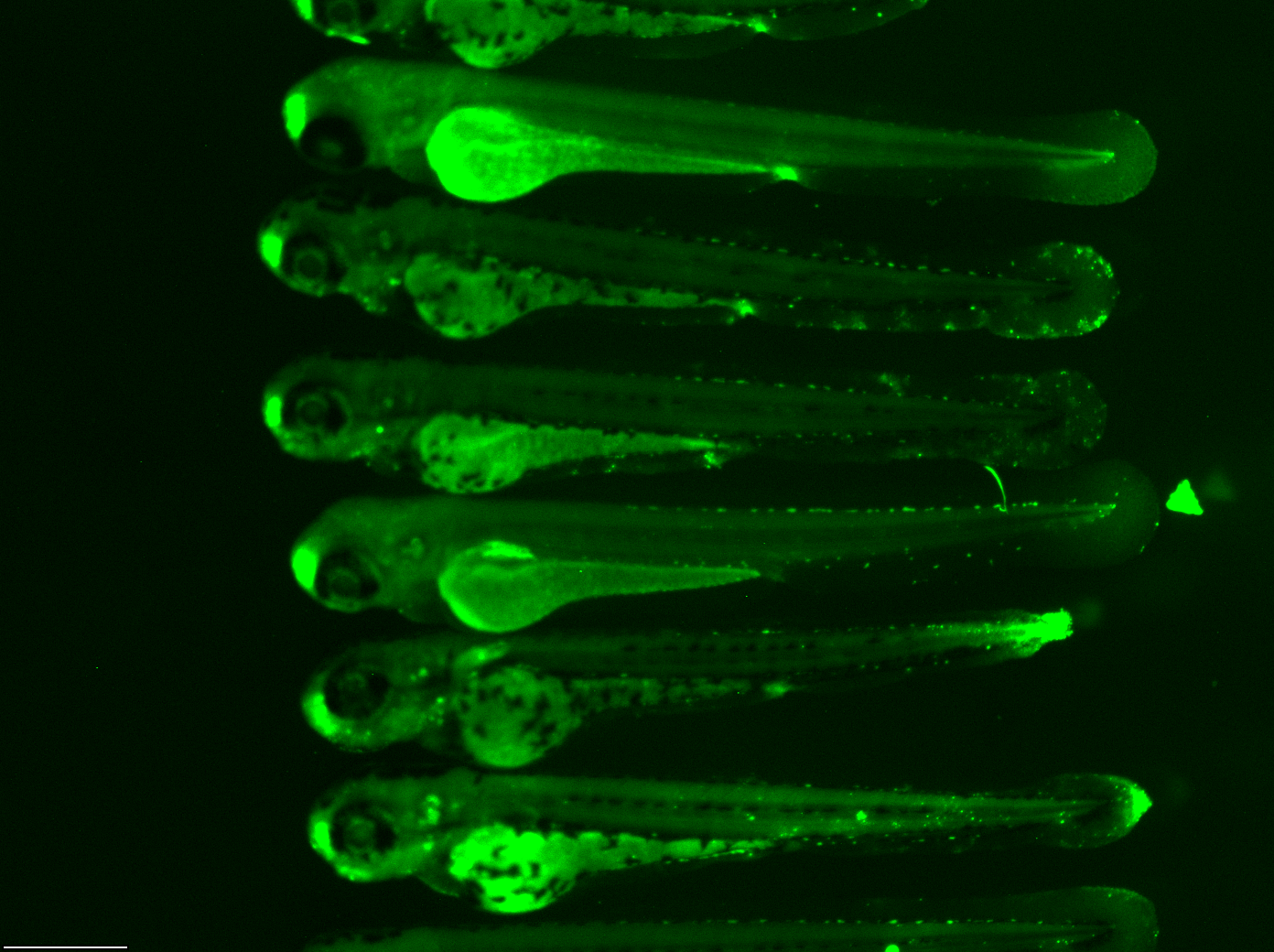

Supplement: Supplementary file 1 [file pharmaceuticals-18-01630-s001.zip › Images Fig. 5F/quercetin_larvae#2.tif]

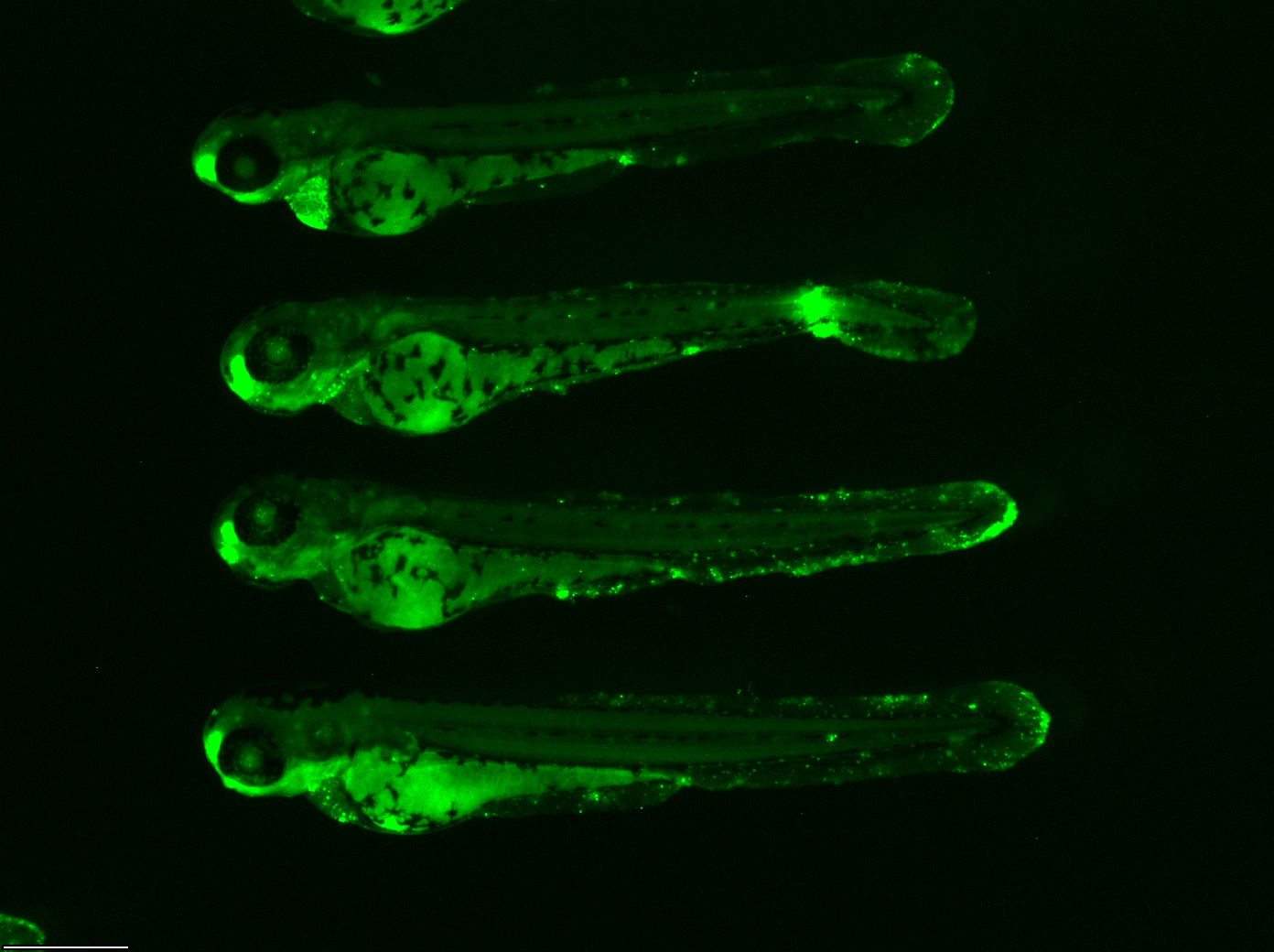

Supplement: Supplementary file 1 [file pharmaceuticals-18-01630-s001.zip › Images Fig. 5F/resveratrol_larvae#4.tif]

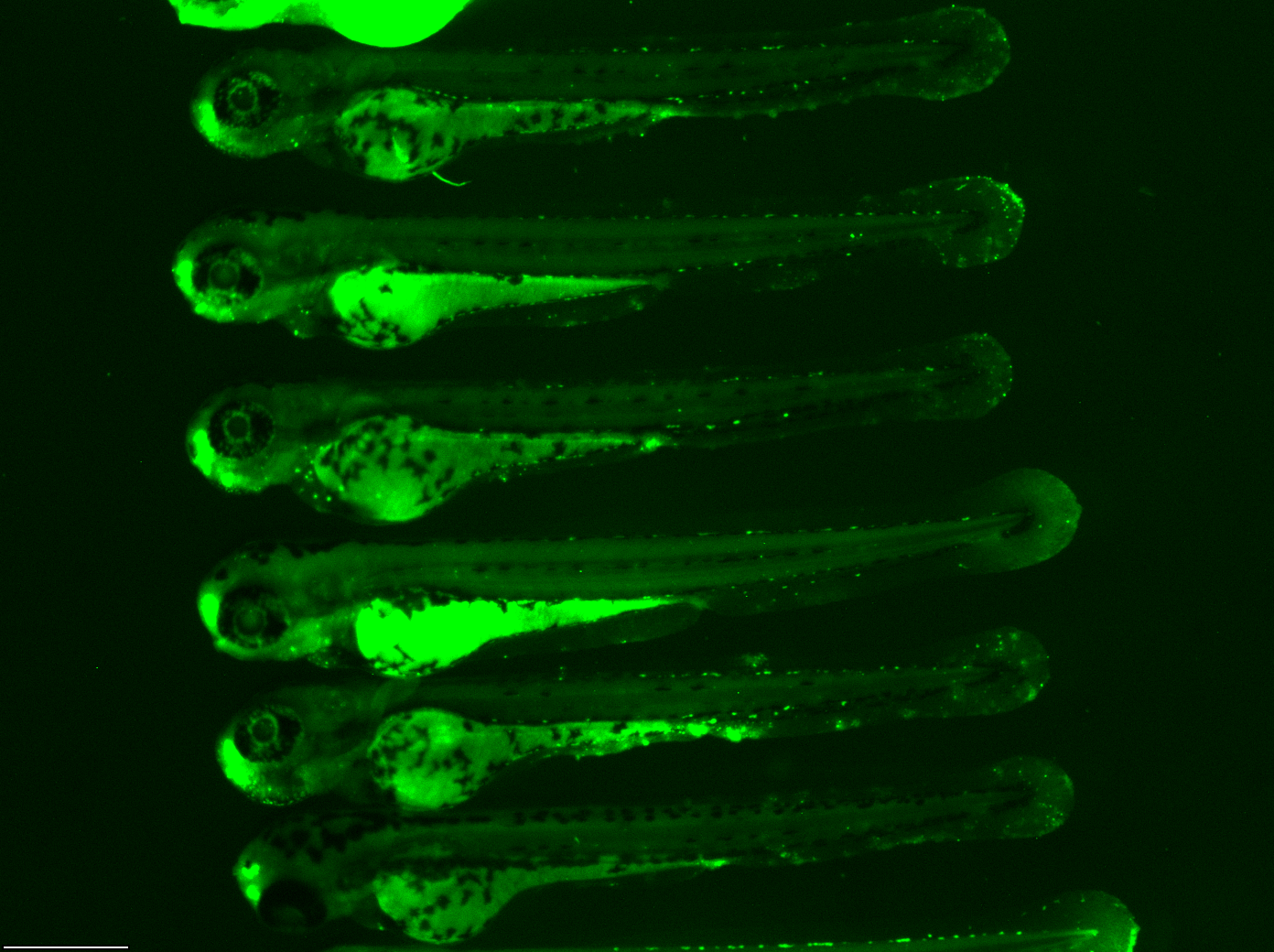

Supplement: Supplementary file 1 [file pharmaceuticals-18-01630-s001.zip › Images Fig. 5F/sakuranetin_larvae#2.tif]

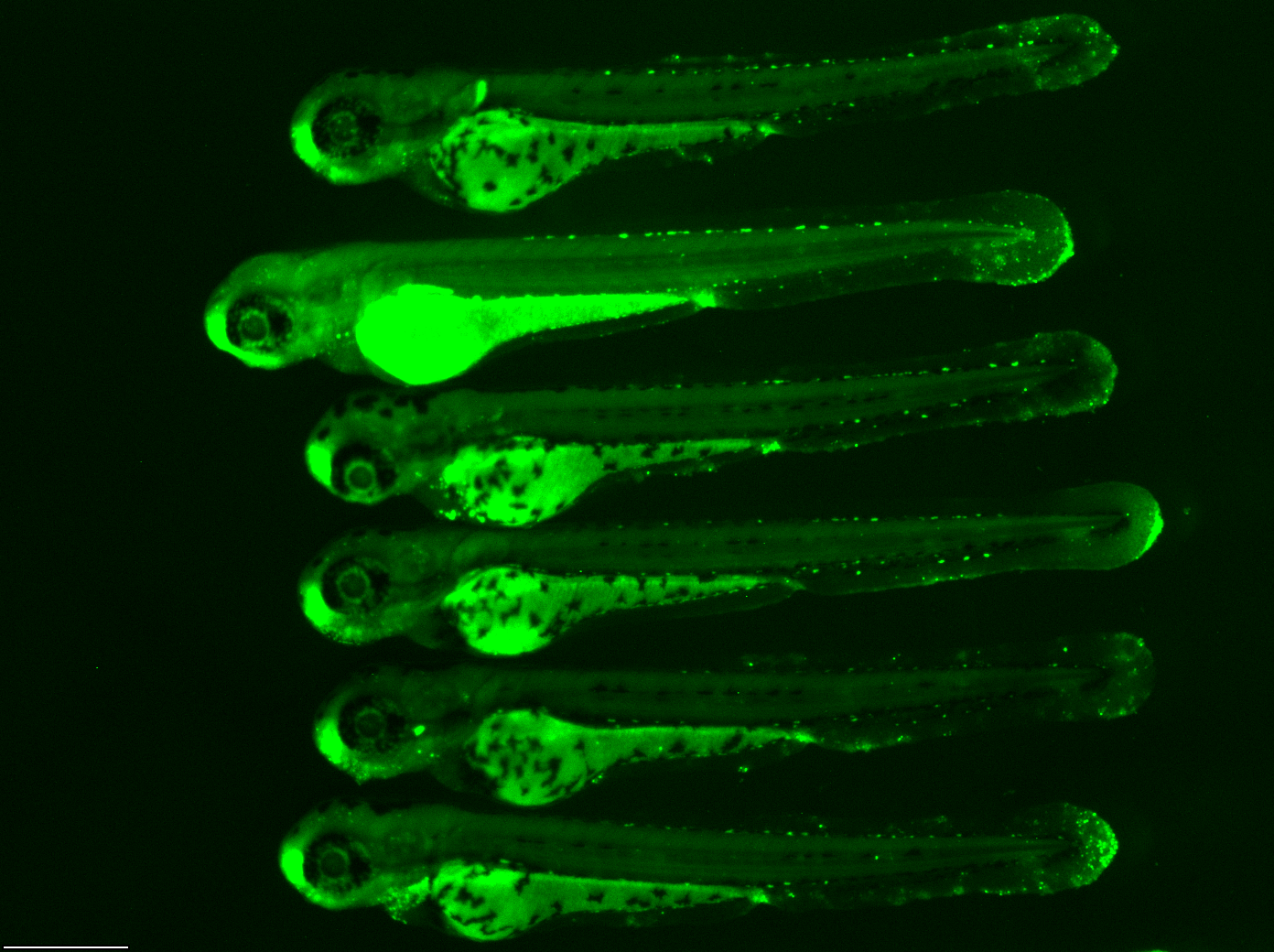

Supplement: Supplementary file 1 [file pharmaceuticals-18-01630-s001.zip › Images Fig. 5F/tricetin_larvae#3.tif]
